# Structural Insights into High Density Lipoprotein: Old Models and New Facts

**DOI:** 10.3389/fphar.2015.00318

**Published:** 2016-01-12

**Authors:** Valentin Gogonea

**Affiliations:** ^1^Department of Chemistry, Cleveland State UniversityCleveland, OH, USA; ^2^Departments of Cellular and Molecular Medicine and the Center for Cardiovascular Diagnostics and Prevention, Cleveland ClinicCleveland, OH, USA

**Keywords:** apolipoprotein A-I (apoA-I), high density lipoprotein (HDL), hydrogen–deuterium exchange mass spectrometry (HDX-MS), small angle neutron scattering (SANS) with contrast variation, electron paramagnetic spectroscopy (EPR), Förster resonance energy transfer (FRET), electron microscopy (EM), molecular dynamics (MD) simulation

## Abstract

The physiological link between circulating high density lipoprotein (HDL) levels and cardiovascular disease is well-documented, albeit its intricacies are not well-understood. An improved appreciation of HDL function and overall role in vascular health and disease requires at its foundation a better understanding of the lipoprotein's molecular structure, its formation, and its process of maturation through interactions with various plasma enzymes and cell receptors that intervene along the pathway of reverse cholesterol transport. This review focuses on summarizing recent developments in the field of lipid free apoA-I and HDL structure, with emphasis on new insights revealed by newly published nascent and spherical HDL models constructed by combining low resolution structures obtained from small angle neutron scattering (SANS) with contrast variation and geometrical constraints derived from hydrogen–deuterium exchange (HDX), crosslinking mass spectrometry, electron microscopy, Förster resonance energy transfer, and electron spin resonance. Recently published low resolution structures of nascent and spherical HDL obtained from SANS with contrast variation and isotopic labeling of apolipoprotein A-I (apoA-I) will be critically reviewed and discussed in terms of how they accommodate existing biophysical structural data from alternative approaches. The new low resolution structures revealed and also provided some answers to long standing questions concerning lipid organization and particle maturation of lipoproteins. The review will discuss the merits of newly proposed SANS based all atom models for nascent and spherical HDL, and compare them with accepted models. Finally, naturally occurring and bioengineered mutations in apoA-I, and their impact on HDL phenotype, are reviewed and discuss together with new therapeutics employed for restoring HDL function.

## Introduction

Decades of research have confirmed that high density lipoprotein (HDL), a plasma cholesterol carrier, is anti-atherogenic and anti-inflammatory in its native state, however it gains atherogenic and pro-inflammatory properties when it becomes dysfunctional via systemic and vascular inflammation (Rosenson et al., 2016). Also, HDL is anti-apoptotic, shields lipids from oxidation, and recovers endothelial function, features that allow HDL to retard atherosclerosis.

While several studies initiated the debate on how strongly plasma HDL levels correlate with cardiovascular disease (CAD; Vergeer et al., 2010), recent advances in understanding the structure of HDL and its multiple physiological functions suggest that chemical modifications of its main protein component, apolipoprotein A-I (apoA-I) by oxidative/nitrating agents generated by myeloperoxidase (MPO), are to a great extend responsible for loss of function and the accumulation of dysfunctional heavily oxidized and crosslinked apoA-I in the artery wall (Smith, 2010; DiDonato et al., 2013, 2014; Huang et al., 2013, 2014; Rosenson et al., 2016). Investigating the susceptibility of apoA-I to oxidative damage at sites of inflammation can help understand how dysfunctional HDL emerges (DiDonato et al., 2013, 2014; Huang et al., 2013, 2014), and should lead to better ways to shield apoA-I from chemical alteration, and to efficient therapeutic approaches for treating atherosclerosis. Overall, the normal physiological activity of HDL rendered dysfunctional, can be resurrected to some extent by therapies with statins and niacin (Lüscher et al., 2014; Rosenson et al., 2016).

Finally, understanding apoA-I oxidative damage requires detailed knowledge of its tertiary structure in both lipid-free and lipid-bound (HDL) forms, and of the remodeling events an HDL particle experiences in its journey from the formation of nascent HDL (nHDL), through the ABCA1 receptor, to the delivery of cholesterol to liver for catabolism and excretion.

This review is not intended to be a comprehensive presentation of published experimental and theoretical studies about the structure and function of HDL. For such information the reader is directed to reviews by Brouillette and Anantharamaiah (1995), Thomas et al. (2008), Phillips et al. (Lund-Katz and Phillips, 2010; Phillips, 2013), and Rosenson et al. (2016) Rather, this paper focuses on reviewing recently published experimental data and theoretical models of lipid free apoA-I and low resolution structures of nHDL and spherical HDL (sHDL) obtained from SANS with contrast variation and isotopic labeling of apoA-I. Changes in HDL phenotype and function upon various naturally occurring and bioengineered mutations in apoA-I, and posttranslational modifications are also reviewed. This review ultimately attempts to evaluate critically how theoretical models of lipid free apoA-I constructed by computational protocols, and HDL models derived from SANS with contrast variation accommodate existing biophysical structural data from alternative approaches.

## Models of lipid free apoA-I

Human apoA-I, a 243 amino acid residue amphipathic protein expressed in liver and intestine (Tall, 1998), binds phospholipids (PL), and free cholesterol (FC) from cell membrane in a process mediated by the ATPase cell receptor ABCA1 (Brunham et al., 2006; Zannis et al., 2006). The first 43 amino acids of the protein primary sequence are encoded by exon three in the apoA-I gene, while exon four encodes the remainder of the primary sequence consisting of 11 and 22 residue repeats (often separated by proline) with amino acid distribution characteristic to an amphipathic α-helix (Segrest, 1977). ApoA-I, the major protein constituent of HDL, is a scaffold for packing lipids (PL, FC, cholesterol ester (CE), triglycerides (TG)); the protein provides thermodynamic stability and physiological functionality to HDL particles in various stages of maturation. Unraveling and understanding the molecular details of its highly dynamic structure is still a challenge and remains a focus for lipoprotein research (Phillips, 2013).

The interaction of lipid-free apoA-I with ABCA1 initiates the translocation of excess FC from periphery cells to liver (the reverse cholesterol transport: RCT). Hence, there is great interest in understanding the molecular details of various steps in RCT like apoA-I/ABCA1 interaction, apoA-I lipidation and dimerization, formation of nHDL and sHDL, HDL binding to the SR-BI receptor, and so forth. For example, the mechanism through which lipid-free apoA-I self-associates in antiparallel fashion within the HDL particle is still unknown. Is the apoA-I monomer or an apoA-I dimer the relevant molecular state for the interaction with ABCA1 or with the cellular membrane? Do apoA-I chains associate in an antiparallel orientation before or after lipidation? A better understanding of the structure and the dynamics of the full length lipid-free apoA-I in solution is needed to answer these questions.

Circular dichroism (CD) measurements on lipid free apoA-I show that about half of the protein chain is in α-helical conformation (Davidson et al., 1996; Gursky and Atkinson, 1996; Saito et al., 2003; Silva et al., 2005a; Jayaraman et al., 2011a). The CD data agree with hydrogen–deuterium exchange mass spectrometry (HDX-MS) studies performed on lipid free apoA-I in solution (Wu et al., 2007; Chetty et al., 2009), which indicate that most of the N-terminus (N_t_) (P_7_–L_44_, T_54_–G_65_, E_70_–Y_115_) has α-helix conformation while the C-terminus (C_t_, E_179_–Q_243_) is in random coil conformation (Chetty et al., 2009).

### Thermodynamics of lipid free apoA-I

Analytical ultracentrifugation measurements indicated that lipid-free apoA-I in solution is actually a mixture of monomers packed as a helical bundle or in an elongated helical hairpin conformation (Rogers et al., 1998a). Gursky et al. (Gursky and Atkinson, 1996) investigated the thermodynamic stability of lipid free apoA-I in solution, and the unfolding from lipid free to lipid bound state by differential scanning calorimetry (DSC). The authors found that lipid-free apoA-I dynamics in solution is characterized by a compact folding state that lacks a definite tertiary structure as would a molten globular state (Freire, 1995, 1997), albeit half of its secondary structure is α-helical (Davidson et al., 1996; Gursky and Atkinson, 1996; Saito et al., 2003; Silva et al., 2005a; Jayaraman et al., 2011a). When binding to lipids, the change in apoA-I conformation is accompanied by an increase in its structural order (the α-helical content increases ~15%) in the same way a native state is more ordered than the molten globular state it originated from. The authors of this study suggest that in plasma, lipid free apoA-I is in a molten globular state when starts to associate with membrane lipids (Gursky and Atkinson, 1996).

Furthermore, the temperature and enthalpy of melting for lipid free apoA-I major conformational transition were found to be independent of concentration; apoA-I is mostly multimeric in the range of concentrations used in this study (1.7–4.7 mg/mL), suggesting that monomer unfolding is detected in the experiment rather than multimer dissociation (Gursky and Atkinson, 1996). This means that hydrophobic interactions between the α-helices of lipid free apoA-I might not contribute substantially to the thermodynamic stability of the monomeric state. In conclusion, the thermodynamic properties of lipid free apoA-I at plasma concentrations should be typical for a molten globular state, i.e., the protein has a compact folding, with a core of α-helices bundled via hydrophobic contacts (Barbeau et al., 1979; Atkinson and Small, 1986; Nolte and Atkinson, 1992; Gursky and Atkinson, 1996), a reduced cooperative unfolding (Reynolds, 1976; Gursky and Atkinson, 1996), lacks a defined tertiary structure, has increased solvent access to its hydrophobic surfaces (Reynolds, 1976; Rosseneu et al., 1977; Gursky and Atkinson, 1996), is easily denatured, has high affinity for hydrophobic ligands, and a predisposition to form aggregates (Reynolds, 1976; Tall et al., 1976).

### Biophysical insights into the structure of lipid free apoA-I

Many biophysical studies concurred that lipid free apoA-I has the ability to easily change conformation and rearrange and exchange when interacting with lipids (Narayanaswami and Ryan, 2000), but at the same time the intrinsic high mobility of certain apoA-I domains (N_t_ and C_t_ termini) hampered any crystallization attempts for either the lipid free or lipid bound forms. To gain insight into apoA-I structure, researchers looked for investigative tools that can gauge to some extent apoA-I's plasticity and the conformational diversity that accompany the transition from lipid free to lipid bound. Earlier studies on apoA-I structure using monoclonal antibodies (Collet et al., 1991; Marcel et al., 1991; Calabresi et al., 1993), ^13^C NMR (Sparks et al., 1992a), and fluorescence spectroscopy (Jonas et al., 1990) revealed that the N_t_ is flexible and changes conformation when apoA-I associates with lipids (Marcel et al., 1991; Sparks et al., 1992a,b; Meng et al., 1993).

However, limitations in the experimental techniques used initially resulted in the characterization of N_t_ of different lengths, which made it difficult to ascertain the role of various apoA-I domains to protein function. For example, the fluorescence experiments produced data about the entire D_1_–W_108_ domain because all tryptophan residues are located in this region and the spectroscopic method cannot assign signals to individual tryptophan residues (Jonas et al., 1990). In another type of analysis, proteolysis studies indicated that an even larger lipid free apoA-I N_t_ domain (D_1_–Y_115_) is resistant to protease cleavage (Brouillette and Anantharamaiah, 1995). These studies concluded that both lipid-free and lipid-bound apoA-I are cleaved at helices 5 and 6 (P_99_–S_142_) while the other helices remain shielded, suggesting that P_99_–S_142_ is a hinged domain (Brouillette and Anantharamaiah, 1995). In the meantime, other researchers succeeded on characterizing smaller domains of the N_t_ (D_1_–A_15_, L_14_–L_90_) by employing monoclonal antibodies that recognize specific apoA-I epitopes (Curtiss and Smith, 1988; Marcel et al., 1991; Bergeron et al., 1995; Curtiss et al., 2006).

In addition, techniques like, protein denaturing (Gursky and Atkinson, 1996; Rogers et al., 1997), circular dichroism (CD; Gursky and Atkinson, 1996; Rogers et al., 1997), surface balance measurements (Phillips and Krebs, 1986; Ibdah and Phillips, 1988; Rogers et al., 1997), fluorescence spectroscopy (Jonas et al., 1990), indicated that the apoA-I N_t_ domain D_1_–N_43_ affects both the stability and the conformation of lipid free apoA-I in solution, and that the full length apoA-I is more stable, and has a different conformation than the truncated form (Δ43–apoA-I). Moreover, it was shown that the domain D_1_–N_43_ is better conserved across different species when compared to C_t_ (A_44_–Q_243_) and it belongs to a different amphipathic α-helix class (G^*^) than the rest of the protein (class A, Y; Segrest et al., 1992). Segrest et al. demonstrated that in a class G^*^ amphipathic α-helix charged residues are distributed randomly on the polar side of the amphipathic α-helix; in a class A, the positively charged residues are located at the polar–non-polar interface, while negatively charged residues aggregate at the center of the polar face; in a class Y amphipathic α-helix the positively charged residues are found both at the polar–non-polar interface and at the center of the polar side (Segrest et al., 1990, 1992, 1994; Jones et al., 1992). In addition of being a different type of amphipathic α-helix, several residues in the D_1_–N_43_ domain are in random coil conformation (Marcel et al., 1991; Nolte and Atkinson, 1992; Segrest et al., 1992, 1994), which would suggest different physiological functions for the N_t_ and C_t_ domains of apoA-I.

### Crystal structures of lipid free apoA-I

**Figure 1:**
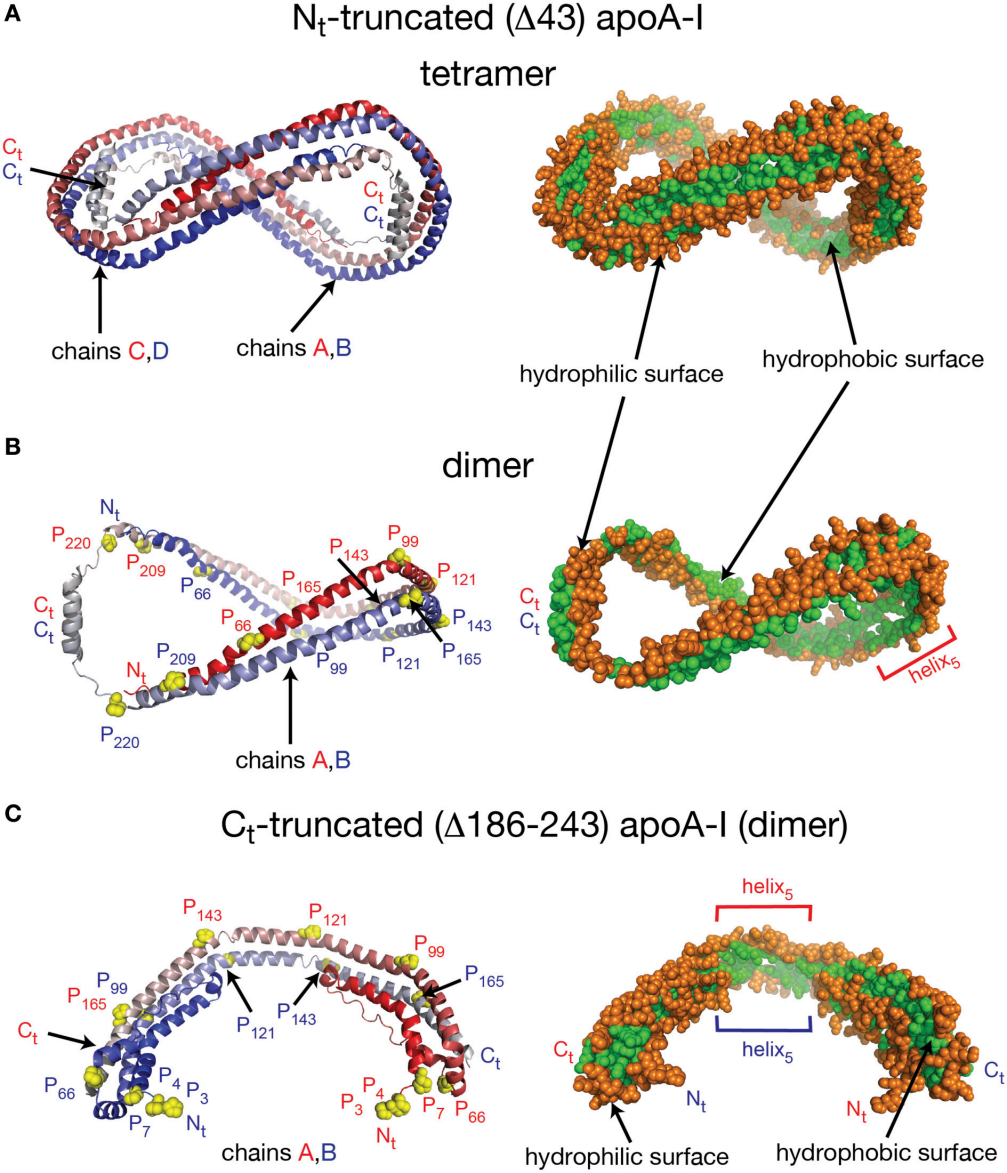
**Crystal structures of truncated lipid free apoA-I**. Both crystal structures have the apoA-I chains orientated antiparallel in helix 5 registry, i.e., the helix 5 domains (P_121_–S_142_) from both chains are on top of each other. **(A)** Left: Cartoon representation of the crystal structure of the Δ43–apoA-I (N_t_ truncated apoA-I) tetramer (PDB id: 1AV1). The structure shows two twisted apoA-I dimers (A,B and C,D) mutually interacting through their hydrophobic surfaces created by the protein chains when in α-helix conformation. The chains in each dimer are colored with gradient red/blue (N_t_ = solid color and C_t_ = faded color). Right: sphere representation of the hydrophobic (green) and hydrophilic (orange) surfaces of the apoA-I tetramer. **(B)** The crystal structure of the Δ43–apoA-I (N_t_ truncated) dimer (PDB id: 1AV1). Left: Proline residues that initiate 11 and 22 residue repeats are shown as yellow spheres. Right: the hydrophilic/hydrophobic surface of the dimer in sphere representation. The C_t_ domains of apoA-I chains have their hydrophobic surface facing outside the closed-loop dimer, while the helix-5 domains have their hydrophobic surface facing inside the dimer. **(C)** Left: Cartoon representation of the crystal structure of the C_t_ truncated form of lipid free apoA-I (1–185; PDB id: 3R2P). The N_t_ is shown to fold back and interact with the rest of the chain. Right: the hydrophilic/hydrophobic surface of the dimer is color coded with green/orange, respectively. The N_t_ domains of apoA-I chains have their hydrophobic surface facing toward the central region of the dimer, while the helix-5 domains have their hydrophobic surfaces facing each other.

Despite the fact that researchers have been unsuccessful in crystalizing the full length apoA-I, two crystal structures of truncated human apoA-I were resolved and published (Borhani et al., 1997; Mei and Atkinson, 2011). The first crystal structure published by Borhani et al. (1997; PDB id: 1AV1) corresponds to an N_t_ truncated mutant of apoA-I that misses the first 43 amino acid residues (Δ43–apoA-I). The unit cell (PDB id: 1AV1) shows a twisted ring tetramer (Figure 1A, left) formed from two apoA-I dimers with apoA-I chains shielding each other's hydrophobic surface by mutual interaction (Figure 1A, right, the sphere representation of the hydrophobic and hydrophilic surfaces of Δ43–apoA-I are colored in green and orange, respectively). The chains in each dimer, colored with gradient red/blue (N_t_ = solid color, C_t_ = faded color), are oriented antiparallel (Figure 1B, left) such that helix 5 of one monomer (P_121_–S_142_) superposes with helix 5 of the other monomer (helix 5 registry, Figure 1B, right). The crystal structure has a high α-helical content (~93%) and part of C_t_, largely hydrophobic (Saito et al., 2004), is α-helical (S_228_–T_242_) and mutually interacts with the same C_t_ segment (S_228_–Q_243_) of the other chain in the dimer (Figure 1B, left). The C_t_ pair of one dimer interacts with helices 5 (P_121_–S_142_) of the other dimer. Proline residues that initiate 11 and 22 residue repeats along the apoA-I chain are shown as yellow spheres (Figure 1B, left). The C_t_ domains of apoA-I chains have their hydrophobic surface facing outside the ring dimer (Figure 1B, right), while the helix-5 domains have their hydrophobic surface facing inside the ring dimer.

Mei and Atkinson (2011) published a second crystal structure corresponding to an apoA-I C_t_ mutant having residues G_185_–Q_243_ deleted (Δ185–243–apoA-I). This crystal structure shows a dimeric apoA-I with chains oriented antiparallel, in which N_t_ folds back to shield part of the hydrophobic surface of the chain (Figure 1C). Like the Δ43–apoA-I mutant, the α-helical content of the C_t_ mutant (80%) is higher than what the CD (Davidson et al., 1996; Saito et al., 2003; Silva et al., 2005a; Jayaraman et al., 2011a) and HDX data (Chetty et al., 2009) for the full length lipid-free apoA-I indicate (~55%). Figure 1C, left shows the cartoon representation of the C_t_ truncated lipid free apoA-I, while the right side displays the hydrophobic and hydrophilic surfaces of the dimer in sphere representation colored with green and orange, respectively. It is interesting to note that the helix-5 domains (P_121_–S_142_), which are not shielded by N_t_ from solvent exposure, have their hydrophobic residues pointing toward each other (Figure 1C, right), a conformation uncharacteristic for apoA-I in HDL.

### Theoretical models of lipid free apoA-I

**Figure 2:**
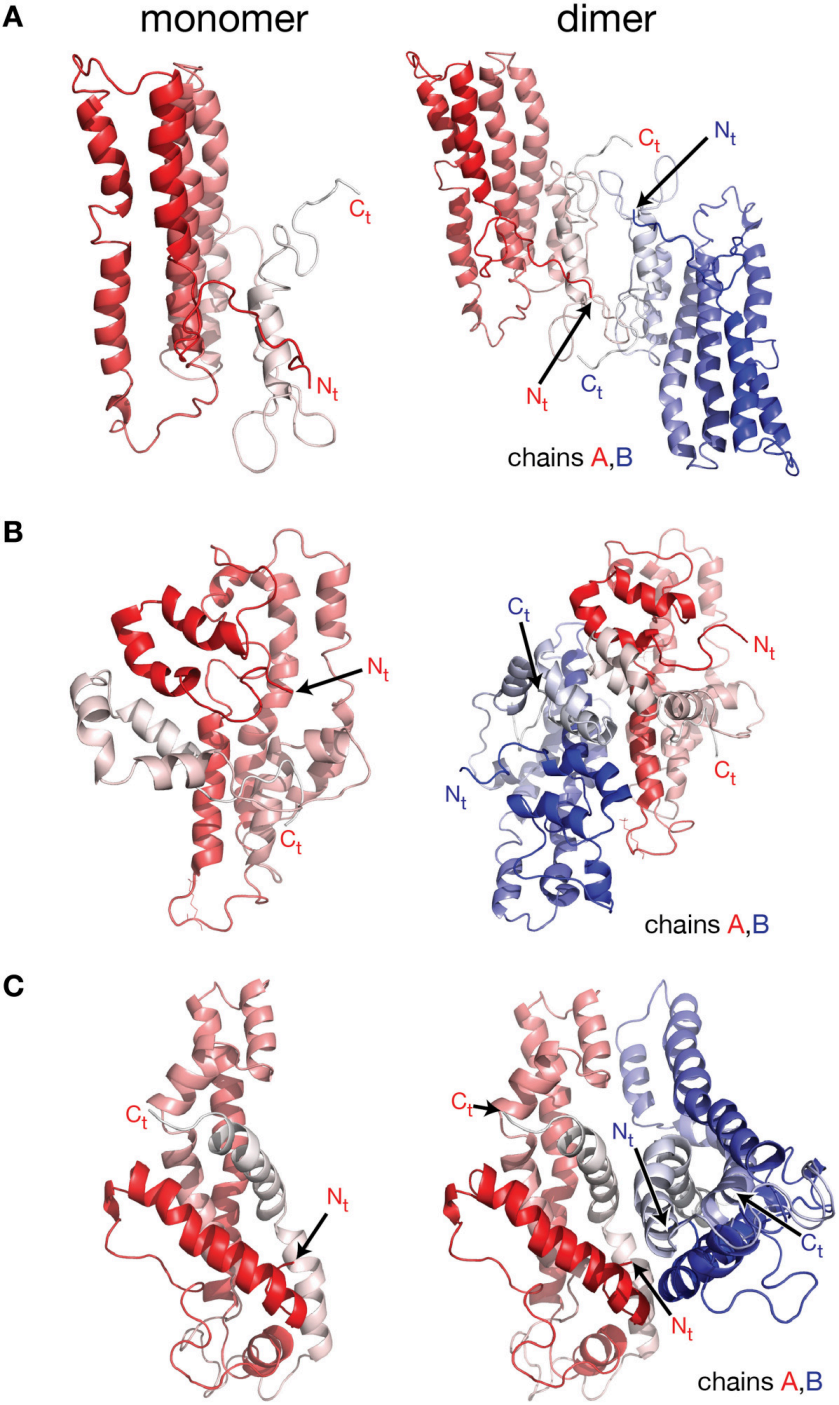
**Molecular models of lipid free apoA-I**. Models of lipid free apoA-I monomer in solution were proposed over the last decade by Silva et al. (2005a), Pollard et al. (2013), and Segrest et al. (2014). **(A)** Left: cartoon representation of the lipid free apoA-I monomer model proposed by Silva et al. (2005a). The protein chain is colored with gradient red (N_t_ is colored with solid color and C_t_ with faded color). Right: cartoon representation of a model of a lipid free apoA-I dimer constructed here by positioning two Silva et al. apoA-I monomer models next to each other such that the C_t_ domains of the two apoA-I chains mutually interact. Most of the reported crosslinks, detected by MS, are between 15 and 35 Å and located at the interaction interface between the two monomers in this model of the dimer. **(B)** Left: cartoon representation of the lipid free apoA-I monomer model proposed by Pollard et al. (2013). Right: cartoon representation of a model of lipid free apoA-I dimer built here from two Pollard et al. apoA-I monomer models. The C_t_ domains of the two apoA-I monomers mutually interact and are oriented perpendicular to each other. The K_88_–K_118_ crosslink detected by MS for the apoA-I dimer is about 10 Å in this dimer model. **(C)** Left: cartoon representation of the lipid free apoA-I monomer model proposed by Segrest et al. (2014). Right: cartoon representation of a model of a lipid free apoA-I dimer constructed here by positioning two Segrest et al. apoA-I monomer models next to each other such that the C_t_ domains of the two apoA-I chains are oriented antiparallel and mutually interact. The K_88_–K_118_ crosslink for apoA-I dimer reported by Pollard et al. (2013) is 58 Å in the dimer model.

The lack of a high resolution crystal structure for the full length protein and the need to understand apoA-I physiological properties like cholesterol efflux, HDL maturation, lipid exchange with cell receptors, etc., stimulated the development of all atom theoretical models for lipid free apoA-I in solution (Figure 2), constructed by incorporating many of the biophysical data gathered from monoclonal antibody, calorimetry (DSC), and limited proteolysis studies, and various geometrical constraints derived from ^13^C NMR, MS-crosslinks, spin coupling (EPR), FRET, and HDX (Jonas et al., 1990; Collet et al., 1991; Marcel et al., 1991; Sparks et al., 1992a; Calabresi et al., 1993; Tricerri et al., 2001; Silva et al., 2005b; Chetty et al., 2009; Jones et al., 2010; Lagerstedt et al., 2012; Pollard et al., 2013). A huge amount of research on lipid free apoA-I was carried out and published in seventies, eighties, and nineties, and there are many publications reviewing them (Brouillette and Anantharamaiah, 1995), however, this review focuses on the presentation in some detail of theoretical models of lipid free apoA-I published in the last decade.

More recent molecular models of lipid free apoA-I were proposed by Silva et al. (2005b), Lagerstedt et al. (2012), Pollard et al. (2013), and Segrest et al. (2014; Figure 2). The molecular models for lipid free apoA-I shown in Figure 2 have the monomeric protein packed as amphipathic α-helices bundled together by hydrophobic interactions (Silva et al., 2005b; Pollard et al., 2013; Segrest et al., 2014). While the three models differ from each other in the location of α-helices along the apoA-I chain, they all comply with various sets of MS-crosslinks. Silva et al. model (Figure 2A, left) was further refined by optimizing its 3-dimensional geometry through energy minimization (Silva et al., 2005b), while Segrest et al. (2014) used molecular dynamics (MD) simulations to validate the thermodynamic stability of their model (Figure 2C, left, *vide infra*). It is worth noting that all three models have part of the C_t_ domain in α-helical conformation, which seems to contradict the protein dynamics data for this region of monomeric lipid free apoA-I (obtained from the HDX-MS experiment; Chetty et al., 2009) that imply a random coil conformation for the C_t_ domain.

Lagerstedt et al. (2012) published the “beta clasp” model of lipid-free apoA-I that incorporates HDX data (Chetty et al., 2009), MS-crosslinks (Silva et al., 2005b), and EPR measurements (Lagerstedt et al., 2012). Interestingly, this is the only molecular model of lipid free apoA-I that has several domains of apoA-I (N_t_, middle domain, and C_t_) in β-strand conformation. The authors suggested that the β-strands might form a hydrophobic core that stabilizes the amphipathic α-helices when the protein is free of lipids and self-associated in solution. The authors hypothesized that the β-strand domains transition to α-helix when lipids bind to apoA-I. The “beta clasp” model of lipid-free apoA-I features a loop centered on residue 139 formed by β-strands pairing. Martin et al. (2006), who identified this loop domain in nHDL (the “looped-belt” model of nHDL (Martin et al., 2006), *vide infra*), claimed that the loop is a key structural feature of apoA-I.

More recently, Segrest et al. (2014) proposed a model for the full length lipid free apoA-I by combining apoA-I domains from the crystal structures of lipid free apoA-I mutants: Δ43–apoA-I (N_t_ truncated; Borhani et al., 1997) and Δ185–243–apoA-I (C_t_ truncated; Mei and Atkinson, 2011), followed by MD simulation on the resultant hybrid model (Segrest et al., 2014). The conditions for the MD simulations were: NPT ensemble, 1 atm (Berendsen's pressure bath), the system was preheated for 50 ps and the simulation was carried out for 30 ns at 500 K. Ewald summation (PME) was used for long range electrostatics and a 12 Å cutoff was employed for van der Waals interactions. The resulting model of lipid free apoA-I was shown by the authors to comply with distant constraints derived from EPR (Lagerstedt et al., 2012), and additional MS-crosslinks identified in the same study (Segrest et al., 2014).

The CD data show that the α-helical content of lipid free apoA-I increases when apoA-I forms multimers at higher concentration (Brouillette and Anantharamaiah, 1995; Gursky and Atkinson, 1996; Jayaraman et al., 2011a) or when apoA-I is bound to lipids, which seems to support the idea that some domain of C_t_ adopts an α-helical conformation when interacting with lipids or when apoA-I self-associates (Phillips, 2013). To distinguish between intra- and inter-chain crosslinks, Silva et al. separated the cross-linked lipid free apoA-I dimer from the monomer by size exclusion chromatography (Silva et al., 2005b). The crosslinks originating from the dimer alone are listed in Table 1 together with distances between the corresponding amino acid residues measured in the models of lipid free apoA-I dimers proposed here (*vide infra*). Table 1 also includes two crosslinks detected by Pollard et al. (2013) in samples of 1 mg/mL, but not in samples of 0.2 mg/mL apoA-I.

**Table 1:** **MS-crosslinks identified in the lipid free apoA-I dimer, and Lys-Lys distances, corresponding to these MS-crosslinks, measured in the model of lipid free apoA-I dimer**.

**MS-xlink^a^**	**Silva et al.^b^**		**Pollard et al.^c^**		**Segrest et al.^d^**	
	**Observed^e^**	**Distance^f^ [Å]**	**Observed**	**Distance [Å]**	**Observed**	**Distance [Å]**
N_t_–N_t_	√	22.4		58.9		12.7
N_t_–K_77_	√	48.1		31.5		39.2
		50.0		31.0		38.1
N_t_–K^g^_118_		22.6	√	52.5		42.3
		24.9		50.8		44.5
N_t_–K_238_	√	16.2		46.7		13.5
		18.0		46.2		17.0
K_88_–K^g^_118_		45.0	√	12.0		55.8
		47.6		9.5		57.0
K_208_–K_208_	√	33.2		36.0		46.1
K_208_–K_238_	√	29.9		47.1		10.7
		31.2		46.0		15.3
K_226_–K_238_	√	29.4		29.4		12.0
		30.1		28.1		13.7
K_238_–K_238_	√	34.8		31.8		29.9

a *Amino acid residues forming the crosslink*.

b *Lipid free apoA-I dimer built from Silva et al. lipid free apoA-I monomer model (Silva et al., 2005a)*.

c *Lipid free apoA-I dimer built from Pollard et al. lipid free apoA-I monomer model (Pollard et al., 2013)*.

d *Lipid free apoA-I dimer built from Segrest et al. lipid free apoA-I monomer model (Segrest et al., 2014)*.

e *The crosslink is checked if it was experimentally observed in the study that reports the model for the lipid free apoA-I monomer*.

f *Minimum distance between the residues involved in the crosslink measured in the lipid free apoA-I dimer model*.

g *This crosslink was reported for an apoA-I concentration of 1 mg/mL but not at 0.2 mg/mL, so it is assumed to form between different apoA-I chains within multimeric lipid free apoA-I (Pollard et al., 2013)*.

By comparing the crosslinking patterns observed in lipid free apoA-I dimer on the one hand, and nHDL, on the other hand, Silva et al. concluded that apoA-I in the lipid free dimer is packed similarly to the lipid free apoA-I monomer and not in an antiparallel fashion as found in nHDL (Silva et al., 2005b). A couple of years later, Lagerstedt et al. (2012) proposed the first molecular model of a lipid free apoA-I dimer based on the “beta-clasp” model of the lipid free apoA-I monomer (*vide supra*). In this apoA-I dimer the apoA-I monomers are aligned according to the location of the spin coupled residues detected by EPR (Lagerstedt et al., 2012) and interact mutually through β-strands domains that mimic the hydrophobic core of nHDL.

To further explore the validity of the lipid free apoA-I monomer models (Silva et al., 2005b; Pollard et al., 2013; Segrest et al., 2014) shown in Figure 2 for the purpose of this review only, models of lipid free apoA-I dimers were constructed by positioning these monomers with respect to each other (Figure 2) such that the reported inter-chain MS-crosslinks (Table 1) are satisfied and the C_t_ of the two apoA-I chains mutually interact. Figure 2A, left shows the model of monomeric lipid free apoA-I proposed by Silva et al. (2005b); the protein chain is colored with gradient red (N_t_ = solid color, C_t_ = faded color). The right panel of Figure 2A shows a cartoon representation of a hypothetical lipid free apoA-I dimer in which most of the reported inter-chain crosslinks (Table 1) are in range 15–35 Å and located at the interaction interface between the two monomers. Figure 2B, left panel shows the cartoon representation of the lipid free apoA-I monomer model proposed by Pollard et al. (2013), and a model of the lipid free apoA-I dimer is shown in the right panel. The mutually interacting C_t_ of the apoA-I chains are oriented perpendicular to each other such that the crosslink K_88_–K_118_ reported for 1 mg/mL apoA-I is satisfied. The distance between residues K_88_ and K_118_ is about 10 Å in this dimer model, but most of the inter-chain crosslinks reported by Silva et al. (2005b; Table 1) are not satisfied in this dimer model. Finally, the left panel of Figure 2C shows the cartoon representation of the lipid free apoA-I monomer model proposed by Segrest et al. (2014) and a model of a lipid free apoA-I dimer at the right. While Segrest et al. (2014) do not specifically report inter-chain crosslinks, to compare this dimer model with the dimer models shown in Figures 2A,B, distances corresponding to the inter-chain crosslinks were measured and listed in Table 1 for this model, too. Many of these inter-chain crosslinks are satisfied in this apoA-I dimer model making it somewhat similar to the dimer model from Figure 2A.

**Figure 3:**
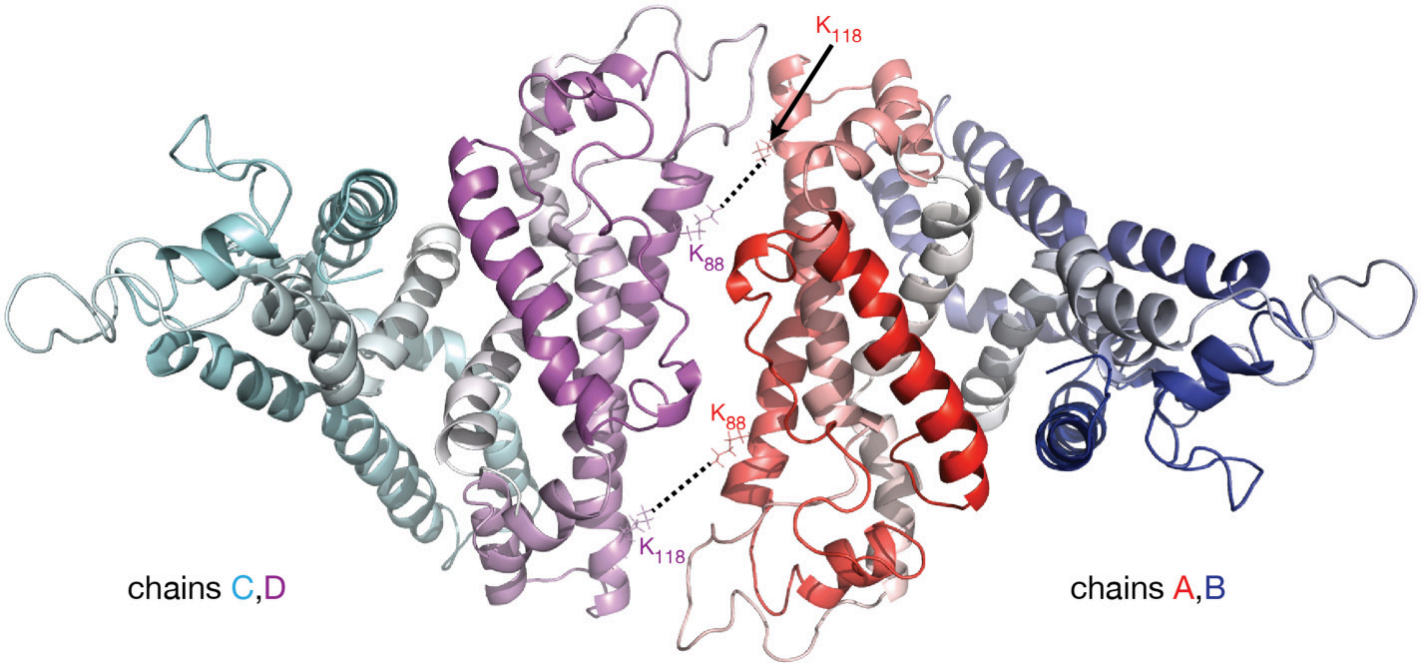
**Molecular model of lipid free apoA-I tetramer**. The figure shows a cartoon representation of a lipid free apoA-I tetramer model constructed from two models of lipid free apoA-I dimer shown in Figure 2C, right (based on Segrest et al. lipid free apoA-I monomer, Figure 2C, left; Segrest et al., 2014). The protein chains are colored with gradient red/blue/cyan/magenta (N_t_ is colored with solid color and C_t_ with faded color). The K_88_–K_118_ MS-crosslink reported by Pollard et al. (2013) at a concentration of apoA-I of 1 mg/mL may come from a tetrameric state of apoA-I. In this hypothetical tetramer model of lipid free apoA-I, the distance between K_88A_ and K_118C_ is 10.6 Å and between K_88C_ and K_118A_ is 9.6 Å.

In the apoA-I dimer models shown in Figures 2A,C the inter-chain crosslinks listed in Table 1 are reasonably satisfied, except for the one between residues K_88_ and K_118_ which has a distance in the range 45–57 Å. This crosslink, reported by Pollard et al. (2013) for 1 mg/mL apoA-I, might actually come from a lipid free full length apoA-I tetramer; in a lipid free apoA-I tetramer model (Figure 3) built from two identical apoA-I dimer models (Figure 2C) the distance between residues K_88_ and K_118_ from mutually interacting apoA-I chains (belonging to different dimers) is about 10 Å. Figure 3 displays a cartoon representation of a lipid free apoA-I tetramer model; the protein chains (A, B, C, and D) are colored with gradient red/blue/cyan/magenta, respectively (N_t_ = solid color, C_t_ = faded color). The distances corresponding to the inter-chain crosslink K_88_–K_118_ are between residues K_88D_ and K_118A_ (K_88A_ and K_118D_) from mutually interacting apoA-I chains D and A belonging to dimers C/D and A/B, respectively. This hypothesis regarding the origin of crosslink K_88_–K_118_ reported for 1 mg/mL apoA-I by Pollard et al. (2013) is supported by the observation made by Silva et al. (2005b) that lipid free apoA-I contains multimers at concentrations as low as 0.1 mg/mL apoA-I.

## Models of nascent HDL

The production of nascent HDL (nHDL) from lipid free/lipid poor apoA-I and cell membrane lipids (PL and FC) by the ABCA1 receptor is the first step in the reverse cholesterol transport (RCT; Zannis et al., 2006). Elucidating the RCT cycle necessitates deep understanding of nHDL structure, unfortunately, the intrinsic highly dynamic nature of apoA-I and difficulties in obtaining homogenous HDL preparations from plasma made the unraveling of defining structural features of nHDL challenging (Thomas et al., 2008; Phillips, 2013). Nonetheless, structural studies on nHDL were greatly facilitated by employing nHDL samples reconstituted from lipid free apoA-I and lipid vesicles (Jonas, 1986). Adsorption of lipid free apoA-I on vesicle's surface facilitates the formation of nHDL (Jayaraman et al., 2011b).

Reconstituted nHDL is still a heterogeneous mixture of particles with sizes between 7.8 and 12 nm, each particle having a particular protein/lipid composition that maximizes protein lipid interaction and ensures thermodynamic stability (Li et al., 2004). Nascent HDL contains two to four apoA-I chains (or a mixture of apoA-I and apoA-II; Cheung and Albers, 1984; Gao et al., 2012) and can accommodate lipid cargos (PL and FC) of various sizes by changes in apoA-I conformation and/or the number of protein chains (Li et al., 2004); a nHDL particle larger than 12 nm is merely formed when more than two apoA-I chains with additional phospholipids are incorporated into the particle (Jonas et al., 1989; Li et al., 2004; Silva et al., 2005b; Vedhachalam et al., 2010).

Many of the early structural studies of nHDL employed particles reconstituted with dimyristoylphosphatidylcoline (DMPC, a shorter saturated acyl chain PL, 14:0), due to its ability to spontaneously form nHDL particles when DMPC vesicles were mixed with apoA-I (Atkinson et al., 1976; Jonas et al., 1977; Tall et al., 1977; Pownall et al., 1978; Swaney, 1980; Matz and Jonas, 1982). Later, the cholate dialysis method (Nichols et al., 1983; Jonas, 1986) was reported to allow reconstitution of nHDL with physiologically relevant PL that have longer and unsaturated acyl chains like palmitoyloleoylphosphatidylcoline (POPC, 16:0–18:1). Reconstituted nHDL particles of various sizes were classified and labeled as a function of the number of apoA-I chains incorporated into the particle and its overall size (Li et al., 2004).

In conclusion, nHDL particles can have multiple compositions including a variable number of apoA-I chains, few lipid types (PL, FC, etc.), and various numbers of lipids per nHDL particle. While this multi-size particle conundrum was early recognized (Brouillette and Anantharamaiah, 1995), the prevalent opinion seemed to be that the double belt model of nHDL (*vide infra*) is sufficient to explain the structural diversity and functional features of nHDL (Phillips, 2013).

### Discoidal models of nHDL

**Figure 4:**
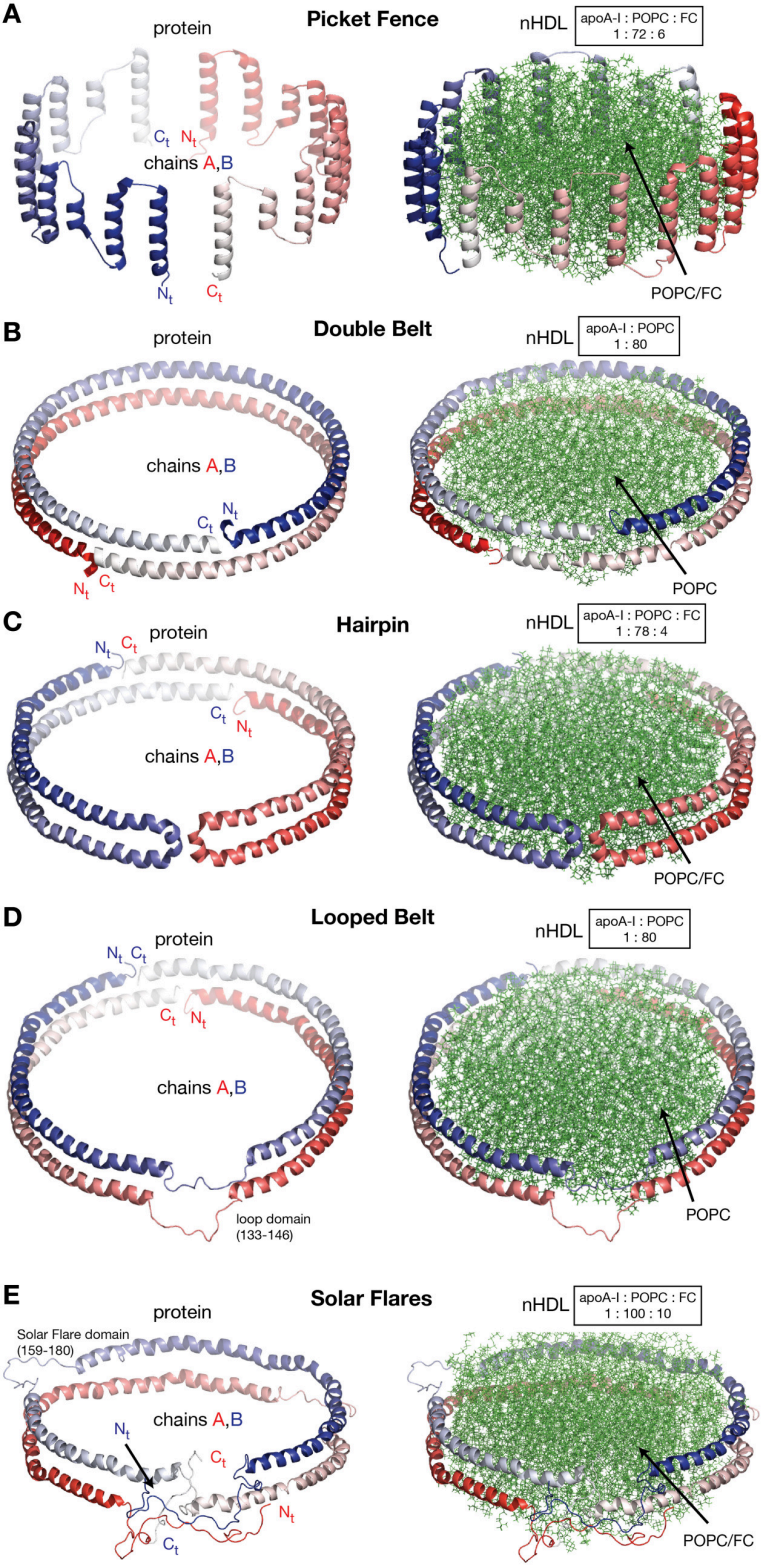
**Discoidal models of nHDL**. In all models the apoA-I chains are shown in cartoon representation and colored with gradient red/blue (N_t_ = solid color and C_t_ = faded color). **(A)** Left: the picket fence model of the apoA-I dimer (Δ43–apoA-I; Jonas et al., 1989; Wald et al., 1990a,b; Nolte and Atkinson, 1992; Phillips et al., 1997); the amphipathic apoA-I α-helices are oriented parallel with the PL molecules. Right: the picket fence model of nHDL (Phillips et al., 1997). The lipid bilayer is made of 144 POPC and 12 FC molecules. **(B)** Left: computational model of the apoA-I dimer (Δ43–apoA-I) used in the Double Belt model of nHDL proposed by Segrest et al. (1999); the apoA-I chains are oriented antiparallel and stacked on top of each other in helix 5 registry. Right: the Double Belt model of nHDL proposed by Segrest et al. (1999). The lipid bilayer is made of 160 POPC molecules. **(C)** Left: model of the apoA-I dimer (Δ43–apoA-I) used in the Hairpin model of nHDL proposed by Silva et al. (2005b); apoA-I monomers form semicircular hairpins located at the periphery of the lipid bilayer, which interact through a couple of residues from repeat 5 and the end domain of C_t_ (V_221_–Q_243_). Right: the Hairpin model of nHDL proposed by Silva et al. (2005b). The lipid bilayer is made of 156 POPC and eight FC molecules. **(D)** Left: model of the apoA-I dimer (Δ43–apoA-I) used in the Looped Belt model of nHDL proposed by Martin et al. (2006); in this model apoA-I features a highly dynamic region (K_133_–E_146_) in random coil conformation that extends half of repeat 5 and the beginning of repeat 6. Right: the Looped Belt model of nHDL proposed by Martin et al. (2006). The lipid bilayer is made of 160 POPC molecules. **(E)** Left: computational model of the full length apoA-I dimer used in the Solar Flare model of nHDL proposed by Wu et al. (2007). Similarly with apoA-I in other belt-type models of nHDL, the apoA-I chains in the Solar Flare model are oriented antiparallel in helix 5 registry. Right: the Solar Flare model of nHDL proposed by Wu et al. (Wu et al., 2007). The lipid bilayer is formed of 200 POPC and 20 cholesterol (FC) molecules. This model of nHDL shows the N_t_ of apoA-I in a globular conformation.

In this review discoidal nHDL refers to models of nHDL in which the protein forms a ring. Experimental evidence produced four decades ago pointed to the fact that nHDL, a macromolecular assembly of amphipathic proteins (apoA-I, apoA-II) and lipids (PL, FC) is made of a lipid bilayer disc with its main protein constituent (apoA-I) located at disc's perimeter (Tall et al., 1976, 1977; Wlodawer et al., 1979; Atkinson et al., 1980; Jonas, 1986). But, the dynamic nature of nHDL, posed insurmountable challenges to crystallization attempts, thus alternative biophysical and biochemical approaches were used to extract structural information about nHDL (Brouillette and Anantharamaiah, 1995; Brouillette et al., 2001). These latter studies lead to early discoidal models of nHDL like the picket fence model (Jonas et al., 1989; Wald et al., 1990a,b; Nolte and Atkinson, 1992; Phillips et al., 1997; Figure 4A), the double belt model (Koppaka et al., 1999; Segrest et al., 1999; Midtgaard et al., 2015; Figure 4B), the hairpin model (Silva et al., 2005b; Figure 4C), and double belt loop models (Maiorano et al., 2004; Bhat et al., 2005; Martin et al., 2006; Wu et al., 2007; Figures 4D,E).

In contrast to lipid free apoA-I, in nHDL, apoA-I is mostly helical; the overall α-helicity of apoA-I was estimated from CD to be ~80% (Jonas et al., 1989; Silva et al., 2005b; Wu et al., 2007). It is worth noting that the 22 and 11 α-helical amphipathic repeats that span most of apoA-I chain (L_44_–Q_243_), are structurally similar but differ in their capabilities of binding lipids (Segrest et al., 2000; Brouillette et al., 2001), which suggests that their contribution to physiological functions of apoA-I (e.g., binding to plasma enzymes and cell receptors) might differ (*vide infra*). These early theoretical models of nHDL describe the particle as having a predominantly α-helical amphipathic apoA-I arranged around the perimeter of a central lipid bilayer with the protein orientation dictated by biophysical constraints, like the hydrophobic and hydrophilic properties along α-helical domains of apoA-I, and geometry constraints between amino acid residues of apoA-I chains obtained from MS-crosslinking (Bhat et al., 2005, 2007; Silva et al., 2005b), Förster resonance energy transfer (FRET; Maiorano et al., 2004), EPR (Martin et al., 2006), and solvent accessibility and dynamics along the polypeptide backbone as probed by hydrogen–deuterium exchange (HDX-MS; Wu et al., 2007; Chetty et al., 2012).

Historically speaking, the picket fence model (Jonas et al., 1989; Wald et al., 1990a,b; Nolte and Atkinson, 1992; Phillips et al., 1997) and the double belt model (Borhani et al., 1997; Rogers et al., 1998b; Koppaka et al., 1999; Segrest et al., 1999; Midtgaard et al., 2015) of nHDL were proposed more than two decades ago. The picket fence model was built from a lipid bilayer with 144 POPC and 12 FC molecules and two apoA-I monomers (D_48_–Q_243_) arranged in head-to-head or head-to-tail dimer configuration oriented similar to how trans-membrane α-helices domains are (Figure 4A, i.e., parallel with the PL molecules in the bilayer); MD simulations and simulated annealing further refined the model (Phillips et al., 1997; Sheldahl and Harvey, 1999). The MD simulation protocol included the use of the CHARMM all atom force field, the TIP3P model of water, and a 10 Å cutoff for van der Waals interactions; the system was first heated up slowly to 500 K and then cooled down fast to 200 K. The picket fence model was popular initially and supported by attenuated total reflection infrared spectroscopy (IR) studies (Wald et al., 1990b). Both the picket fence and the double belt models are consistent with early small angle neutron scattering (SANS) with contrast variation measurements (Atkinson et al., 1980) that supported a bilayer arrangement of the lipid and a circumferential apoA-I.

The publication of the crystal structure of the N_t_ truncated lipid free apoA-I (Borhani et al., 1997; Figure 1A) revived the interest in the double belt model, which incorporated the newly discovered structure of the antiparallel apoA-I dimer and a lamellar lipid (160 PL) extracted from a POPC bilayer thermodynamically stabilized by MD simulations (Heller et al., 1993). Subsequently, the double belt model quickly captured renewed interest from many researchers who used a variety of biophysical approaches to support it (Koppaka et al., 1999; Maiorano et al., 2004; Bhat et al., 2005, 2007; Silva et al., 2005b; Martin et al., 2006). The double belt model of nHDL gained further acceptance as its overall shape was consistent with images of discoidal nHDL obtained from earlier EM studies that showed nHDL particles stacking as coins (a.k.a. “discoidal” nHDL; Forte et al., 1971; Tall et al., 1977), while MD simulations were used to demonstrate the thermodynamic stability of the model (Klon et al., 2002; Catte et al., 2006, 2008; Jones et al., 2009; Gu et al., 2010). Similar images of nHDL were obtained later by cryo-EM (Zhang et al., 2011; Zhang and Ren, 2012) and atomic force microscopy measurements (Gan et al., 2015). In the first MD simulation (1 ns) of the double belt model (Klon et al., 2002) the protocol included a simulation temperature of 300 K, the use of CHARMM22 force field for protein and CHARMM24 for lipids, van der Waals interactions were truncated beyond 10 Å, a spherical boundary potential was applied to prevent solvation shell water molecules from leaving the droplet. Over the years many researchers came to support the double belt model, which became the entrenched view of nHDL structure (Lund-Katz and Phillips, 2010; Phillips, 2013).

Figure 4B shows at the left the ring model of the apoA-I dimer (Δ43–apoA-I) used by Segrest et al. (1999) to build the double belt model of nHDL. The N_t_ truncated apoA-I chains (Δ43–apoA-I; colored with gradient red/blue) are oriented antiparallel and stacked on top of each other in helix 5 registry. At the right, the double belt model of nHDL is displayed in which two truncated apoA-I chains wrap around a lipid bilayer formed from 160 POPC molecules; the interior hydrophobic surface of apoA-I ring interacts with the acyl chains of the lipid.

Figure 4C shows an alternative discoidal model of nHDL proposed by Silva et al. (2005b) in which apoA-I chains are not mutually associated with each other, but rather form separate hairpins circumferentially positioned around the lipid bilayer and interacting with each other through a couple of residues from helix 5 and the C_t_ domain, V_221_–Q_243_. The hairpin and the double belt models are practically indistinguishable as far as geometrical constraints are concerned, but the double belt model gives nHDL stronger structural integrity as the two apoA-I chains are interlocked through salt bridges made by residues carrying opposite charges (Segrest et al., 1999).

While CD experiments confirmed a high degree of α-helicity (>70%) for apoA-I in nHDL (Jonas et al., 1989), several studies revealed that certain regions of apoA-I are highly dynamic and last long enough in random coil conformation to be detected through biophysical techniques such as EPR and HDX. These studies lead to a class of discoidal models coined the “looped-belt” models of nHDL (Maiorano et al., 2004; Bhat et al., 2005; Martin et al., 2006; Wu et al., 2007). For example, Martin et al. (2006) used EPR to identify a highly dynamic region of apoA-I that extend half of helix 5 and the beginning of helix 6 (Figure 4D, K_133_–E_146_). Lagerstedt et al. hypothesized recently that this region of apoA-I (K_133_–E_146_) in random coil conformation is inherited from lipid free apoA-I and may be a key functional domain of apoA-I (Lagerstedt et al., 2012).

A more recent looped-belt model of nHDL obtained by incorporating biophysical constraints derived from hydrogen–deuterium exchange mass spectrometry (HDX-MS) and MS-crosslinking data is the Solar Flares model (Wu et al., 2007). Figure 4E shows at the left the full length apoA-I dimer used in the Solar Flares model. Similarly to apoA-I in the double belt model, the apoA-I chains in the Solar Flares model are oriented antiparallel in helix 5 registry and form a ring. The Solar Flares model of nHDL is shown in the right panel of Figure 4E, in which the lipid bilayer was built with 200 POPC and 20 FC molecules; this model of nHDL has 60 lipids more than the double belt model and represents a physiologically relevant nHDL particle as cholesterol was added to the lipid phase and lipid free N_t_ of apoA-I were attached in globular conformation.

The Solar Flares and the other belt type models of nHDL (Figures 4B–D) have both similar and distinctive features. First, all belt type models have two apoA-I chains oriented antiparallel, in helix 5 registry, and form a ring. Second, their lipid phase is a rather planar lipid bilayer circumscribed by the protein. Third, the models accommodate the majority of distance constraints derived from MS-crosslinking (Segrest et al., 1999; Silva et al., 2005b; Wu et al., 2007). The distinctive features are: the Solar Flares model uses the full length apoA-I with the N_t_ of apoA-I in a globular conformation while the other belt type models use the N_t_ truncated apoA-I (Δ43–apoA-I). In addition, the conformations of apoA-I chains in the Solar Flares model accommodate solvent accessibility and dynamics constraints derived from HDX-MS data (Wu et al., 2007). Another distinctive and rather controversial feature (Shih et al., 2008) of the Solar Flares model is the presence of the Solar Flares domains (L_159_–A_180_) predicted to have random coil conformation with dynamics restricted by three-way salt-bridges (R_160_–H_162_–D_168_). These apoA-I domains predicted to bind to lecithin cholesteryl acyltransferase (LCAT), the enzyme that matures nHDL into sHDL (Wu et al., 2007), were constructed in random coil conformation to accommodate the HDX-MS data (Wu et al., 2007).

Salt-bridges (R_160_–H_162_–D_168_) in the Solar Flares loops were observed to form during energy minimization calculations performed on the model (Wu et al., 2007). Other researchers used MD simulations to dispute this feature of the Solar Flares model by showing that the salt bridges collapse after 1 ns (Shih et al., 2008). In a subsequent study, Gogonea et al. (2010) used MD simulations to reinforce the idea that the salt bridges in the Solar Flares regions of apoA-I are persistent, breaking, and reforming during the simulation (>80 ns; Gogonea et al., 2010). The MD simulation protocol used in this study included: NVT ensemble, simulation temperature 300 K, Berendsen thermostat, Ewald summation (PME), 10 Å cutoff for van der Waals interactions, GROMOS87 force field, and the SPC water model. In retrospect, it seems plausible to consider structural features like salt bridges as transient constructs that dynamically form and break. While theoretical calculations can lead to artifacts in molecular configurations due to simplifications in the molecular mechanics force fields used to describe molecular interactions, the experimental HDX-MS measurements on nHDL indicate that the Solar Flare regions of apoA-I retain a random coil conformation on a time scale of minutes (Wu et al., 2007).

In brief, while inheriting many features of nHDL belt type models, the Solar Flares model has its own merits. It is the first all atom (three dimensional) model of nHDL to use full length apoA-I, includes information about solvent accessibility and dynamics of apoA-I with near residue resolution (achieved through computational manipulation of the entire set of overlapping peptides produced by the HDX/peptide digestion-MS experiment, Wu et al., 2007), and incorporates a key functional feature of nHDL, i.e., the location and conformation of apoA-I domains (Solar Flares regions) that bind LCAT and facilitate nHDL maturation.

Despite the fact that belt type models of nHDL incorporate a wealth of biophysical data, share an antiparallel orientation of two apoA-I chains and an overall planar conformation of the apoA-I double chain, none of these models is based upon direct visualization of the shape of apoA-I or the lipid core within nHDL in solution. Thus, the overall conformation of the apoA-I dimer within the particle is still debated (Wu et al., 2009; Gogonea et al., 2010, 2013; Lund-Katz and Phillips, 2010; Phillips, 2013).

### Models of nHDL derived from SANS with contrast variation

#### The use of SANS with contrast variation in structural biology

Small-angle scattering (SAS), a technique that rely on scattering X-rays or neutrons off matter in liquid or solid form, was employed to investigate the structure of biomolecules in solution for many decades (Guinier, 1938). The neutrons used in SANS experiments have low energy (with wavelength in the range of 4–6 Å) and do not alter the conformation of biomolecules during scattering in contrast to the (high energy) X-rays employed in SAXS experiments. This particular characteristic of the neutrons makes them excellent probes for exploring the structure of macromolecular complexes. Signal averaging due to molecular rotation in solution, casted SAS as a low-resolution structural technique in the sense that it does not produce atomic coordinates, but rather provides information about the size and the overall shape of a macromolecular system (Neylon, 2008). Availability of monodisperse samples is crucial for the accurate interpretation of the scattering data in terms of structural parameters; therefore sample preparation and assessment of monodisperity are critically important factors for SAS structural studies. In early studies only simple structural features, like the radius of gyration, were derived from SAS data, but, over time, the analysis of scattering intensity grew in sophistication leading eventually to computer programs (Svergun et al., 1995) that can nowadays de-convolute the scattering intensity into a distance distribution function, *P*(*r*) (Svergun, 1992), and use the latter to reconstruct a low resolution structure of the molecular system (Svergun, 1999; Svergun and Koch, 2003; Jacques and Trewhella, 2010).

A significant advance in SAS was the development of SANS with contrast variation, a more sophisticated technique involving isotopic labeling of biomolecules and the use of D_2_O to achieve the contrast. In a SANS with contrast variation experiment a component (e.g., protein, lipid, DNA, RNA) in a macromolecular complex is “masked” by changing the D_2_O-to-H_2_O ratio in the buffer until the scattering length density of the latter matches that of the masked component. This ability to map the position of individual components in a macromolecular complex in solution makes SANS a rather unique technique capable of connecting structural and stoichiometric information of rather big macromolecular complexes in a way that is difficult to achieve by other methods.

SANS with contrast variation was very useful in determining the arrangement of components within a bimolecular complex. For example, two decades before the crystal structure of the nucleosome was resolved (Luger et al., 1997), SANS with contrast variation predicted the structural orientation of the DNA and protein components within the fundamental unit of chromatin (Pardon et al., 1975), and correctly located the protein and RNA subunits within the ribosome in the 30S subunit (Capel et al., 1987) and 50S subunit (May et al., 1992). In other studies, SANS with contrast variation was used to investigate the structure of T-cell surface glycoprotein CD1d1 in complex with β-2 microglobulin (Schiefner et al., 2009), and the protein kinase R (a key player in the interferon pathway, VanOudenhove et al., 2009) known to contain three folded regions separated by disordered linkers (Lemaire et al., 2006). Yet, in another SANS with contrast variation study of inhibitor binding (Sda, Whitten et al., 2007 and KipI, Jacques et al., 2008) to histidine kinase (KinA, *Bacillus subtilis*), a reduction in the size of the KinA dimer and an apparent enlargement of inhibitor size, due to two inhibitors binding on opposite sides of the KinA dimer, were detected. The study showed that inhibitors of KinA do not bind to the flexible hinge connecting the catalytic and self-association regions of KinA (as previously thought; Rowland et al., 2004), but rather at the base of the KinA self-association domain. The structural insight obtained from SANS with contrast variation enthused new mutagenesis studies (Cunningham and Burkholder, 2009) that revealed both autokinase and phosphotransferase reactions to be inhibited by KipI and Sda. The SANS prediction of the Sda binding site on KinA was later confirmed by the crystal structure of the complex of KinB (a homolog of KinA from *Geobacillus stearothermophilus*) with Sda (Bick et al., 2009).

#### SANS low resolution structures and models of nHDL

Before delving into presenting more recent nHDL models derived from SANS with contrast variation experiments, it may be useful to recount that in general lipoproteins (macromolecular assemblies of proteins and lipids) contain molecules with different neutron scattering properties making them excellent candidates for structural studies employing SANS with contrast variation. For example, the protein and the phospholipid heads scatter neutrons (at zero scattering angle) as buffer solutions of 42% D_2_O and ~30% D_2_O, respectively (Serdyuk et al., 2007). On the other hand, the lipid acyl chains are less dense and have a higher hydrogen content, thus scattering neutrons as a buffer solution of ~5% D_2_O (Serdyuk et al., 2007). Because a lipid molecule is composed of domains with different neutron scattering properties (lipid head vs. tail), as a whole, a phospholipid exhibits neutron scattering properties as a solution with 12% D_2_O (Serdyuk et al., 2007).

Nearly four decades ago, Atkinson et al. reported small angle X-ray scattering (SAXS; Atkinson et al., 1976) and SANS with contrast variation studies (Atkinson et al., 1980) on nHDL reconstituted with DMPC. The authors have not published a low resolution structure of nHDL at that time, but their findings supported the idea that the shape of nHDL is oblate, the lipid is bilayer and the protein is circumferential to the lipid within the particle, and stated that their SAXS and SANS data are consistent with the discoidal model of nHDL. Additional small angle X-ray scattering (SAXS) studies on reconstituted nHDL and MD simulations (Denisov et al., 2004; Shih et al., 2007; Jones et al., 2009) lent further credence to the discoidal model.

Preliminary SANS experiments on nHDL particles reconstituted with the wild type protein revealed that the difference in neutron scattering properties of the protein and the lipid (42 vs. 12% D_2_O) is not enough to produce a well-resolved low resolution structure for the protein (Wu et al., 2009). To enhance the contrast (difference in scattering properties) between the protein and the lipid, the protein is labeled through deuterium enrichment by expressing it in media containing D_2_O (Wu et al., 2009). The buffer solution matches the scattering properties of a deuterium-enriched protein when it has ~92% D_2_O and the contrast between protein and lipid (given in %D_2_O) increases from 30 (= 42–12) to 80 (= 92–12); the scattering intensity increases with the square of the contrast (Svergun and Koch, 2003).

#### The double superhelix model of nHDL

**Figure 5:**
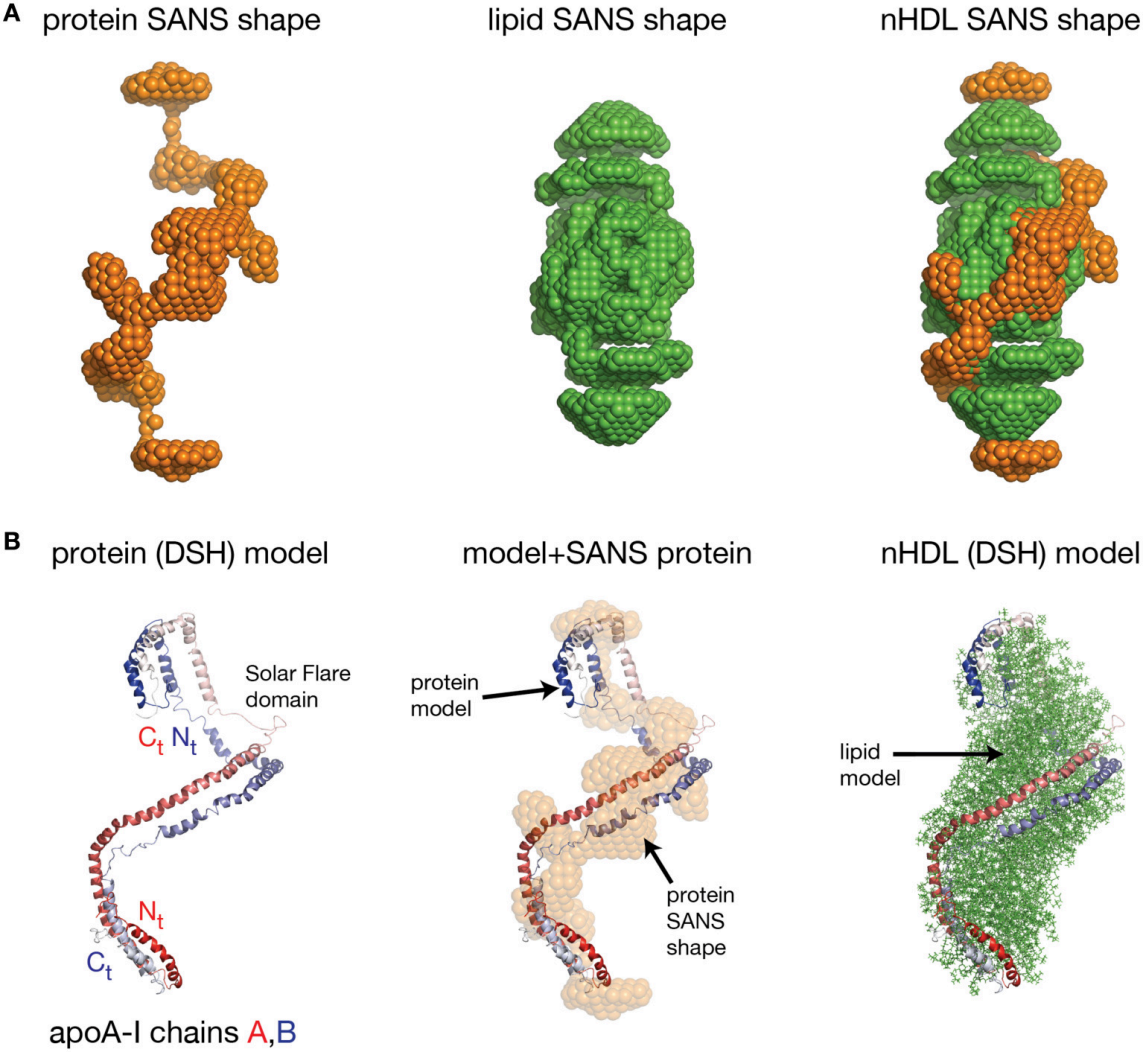
**The low resolution structure and the DSH model of nHDL reconstituted with POPC and FC**. **(A)** Left: The low resolution structure of the protein component of nHDL. Middle: The low resolution structure of the lipid component of nHDL. Right: The low resolution structure of nHDL as a combination of the low resolution structures of the protein and lipid components of nHDL. **(B)** Left: the DSH model of the full length apoA-I dimer in nHDL proposed by Wu et al. (2009). The apoA-I chains shown in cartoon representation and colored with gradient red/blue, are oriented antiparallel in helix 5 registry. The Solar Flares domains introduced in the Solar Flares model of discoidal nHDL were retained in this model of nHDL to accommodate the HDX-MS data. Middle: Superposition of the spiral apoA-I double chain with the low resolution structure of the protein component of nHDL obtained by SANS with contrast variation. Right: The DSH model of nHDL; the lipid model was built from 200 POPC and 20 FC molecules in a combined micellar/lamellar phase. The lipids in close proximity to protein follow a lamellar arrangement while those further away are arranged radially (as in a micelle) in order to accommodate the open conformation of apoA-I and avoid exposing acyl chains to solvent.

Wu et al. obtained the first low resolution structure of nHDL by employing SANS with contrast variation and isotopic labeling of the protein component (Wu et al., 2009). The nHDL particles used in this study (prepared with the cholate dialysis method Jonas, 1986) were 11 nm in diameter measured by light scattering (9.6 nm from ND-PAGE gel), and contained two apoA-I chains, 210 POPC and 15 FC per nHDL particle (Wu et al., 2009); an nHDL particle with a similar size and composition was reported by Marcel and coworkers (Calabresi et al., 1993; Meng et al., 1993). Wu et al. reported low resolution structures both for the protein and the lipid components of nHDL (Wu et al., 2009), and the low resolution structure for the protein component, shown in the left panel of Figure 5A, was obtained from measurements of nHDL particles reconstituted with deuterium-enriched apoA-I and dialyzed in a buffer solution with 12% D_2_O. At this concentration of D_2_O the lipid phase and the buffer have the same scattering properties, so the lipid phase becomes “transparent” and the difference in scattering signal (between sample and buffer) is due to the protein alone. The low resolution structure of apoA-I in this nHDL particle turned out to be an open spiral (Wu et al., 2009). A second measurement of nHDL particles reconstituted with non-labeled apoA-I and dialyzed in a buffer solution with 42% D_2_O provided the low resolution structure for the lipid phase of nHDL, which has an ellipsoidal (prolate) shape (Figure 5A, middle). The low resolution structure of nHDL was obtained by combining the low resolution structures for the protein and the lipid components (Wu et al., 2009; Figure 5A, right).

Further, the authors used geometrical constraints derived from other biophysical methods like MS-crosslinking, FRET, EPR, HDX-MS, particle composition analyses, to build a molecular model that could fit two full length apoA-I chains into the low resolution structure of the protein component (Figure 5B, left), and 200 PL (POPC) and 20 FC molecules into the low resolution structure of the lipid component (Figure 5B, middle). The combined low resolution structure of the protein and lipid was used to determine the orientation of PL and FC molecules such that the hydrophobic surface of the protein is in contact with the acyl chains of the lipids. This requirement together with the open conformation of the protein lead to a lipid phase model with combined lamellar–micellar arrangement and not a pure bilayer as in discoidal nHDL. The combined molecular model for the protein and lipid was coined the Double Superhelix model (DSH); Figure 5B shows at the left the spiral conformation of the apoA-I double chain, in the middle the overlap between the all atom model and the low resolution structure of the protein component, and the DSH all atom model of nHDL at the right.

The DSH model has many common features with the discoidal models of nHDL, but at the same time has several distinctive new characteristics. Like the discoidal models of nHDL, the DSH model incorporates two apoA-I chains per nHDL particle associated in antiparallel orientation in helix 5 registry. The two apoA-I chains, made to a large extent of amphipathic α-helices, define a hydrophobic surface facing and binding the lipid acyl chains. The DSH model accommodates all MS-crosslinks, EPR, and FRET distance constraints reported for nHDL and confirmed in the double belt and Solar Flares models as well. The phospholipid molecules are oriented such that their heads are on particle's surface while their tails are inside the particle interacting with one another and with the protein hydrophobic surface. Like the Solar Flares model, the DSH model was constructed with full length apoA-I chains.

The most distinctive feature of the DSH model is the open shape of the apoA-I double chain, whose conformation follows the experimentally determined low resolution structure of apoA-I in nHDL (Figure 5B, middle). That is, the protein belt made of two antiparallel oriented apoA-I chains does not form a ring as in discoidal models. The open conformation of the protein implies the existence of regions of the lipid phase, which are not bilayer but rather monolayer with radial orientation of PL like in micelles. However, the PL in close proximity to the protein adopts a bilayer arrangement as in discoidal models because of their interaction with the protein inner hydrophobic surface and the requirement that the PL polar head groups remain on the particle surface. It is important to emphasize that three dimensional discoidal models are not based on direct visualization of an nHDL particle, but they are rather computational-theoretical models that incorporate geometrical constraints derived from various biophysical techniques (MS-crosslinking, FRET, ESR, HDX-MS, etc).

Despite its many similarities with the discoidal models of nHDL, the DSH model was met with resistance and skepticism. For example, Jones et al. (2010) referred to the DSH model as “dramatically different from the standard model” and questioned its validity through the use of MD simulations (65 ns; Jones et al., 2010) concluding that the DSH model is not thermodynamically stable. In addition, these authors showed by FRET that the distance between residues 40 and 240 of apoA-I in nHDL is in the range of 28–34 Å (Jones et al., 2010) and cannot be accommodated by the DSH model. In another computational study of the DSH model (using a different simulation temperature and ensemble), Gogonea et al. (2010) used also MD simulation (>80 ns) to investigate the thermodynamic stability of the DSH model and found that the model is thermodynamically stable for tens of nanoseconds (Gogonea et al., 2010), the apoA-I chains retain the open conformation (spiral shape) and the lipid phase remained a lamellar–micellar mixture. While the elongated lipid phase ellipsoid (prolate) changed somewhat into a spheroid, the most distinctive features of the model were preserved during the entire simulation. The overall change in the shape of the particle might be attributed to the simplified molecular mechanics force fields used to describe molecular interactions in MD simulations (Gogonea et al., 2010).

In addition, Gogonea et al. (2010) showed that following MD simulation, the conformations of the two Solar Flares regions in the DSH model (LCAT binding domains of apoA-I) are not identical raising the possibility that only one site for binding LCAT in nHDL is functional, a fact that can impact nHDL maturation. For example, the discoidal models create a barrier for FC diffusion as the protein ring separates the lipid phase into two disjoint lipid surfaces and impedes the diffusion of FC from one surface to another. On the other hand, the DSH model presents a continuous lipid surface due to the open conformation of apoA-I and the micellar-lamellar arrangement of the lipid phase, thus allowing free diffusion of FC, which can be esterified at a single functional LCAT binding site.

Jones et al. noted that the DSH model couldn't accommodate a FRET geometry constraint between residues 40 and 240 of apoA-I because the apoA-I termini are far apart due to its spiral conformation. While this is true in the DSH model, Gogonea et al. argued that the distance constraint between residues 40 and 240 of apoA-I can be accommodated in a SANS model of nHDL reconstituted with DMPC (~80 DMPC per apoA-I chain; Gogonea et al., 2013). While, Jones et al. have not specified the composition of the nHDL preparations used in their FRET study, it is possible that the nHDL particles employed by Jones et al. to differ in lipid composition (and size) from those used to derive the DSH model.

#### Low resolution structures of nHDL reconstituted with DMPC and FC

**Figure 6:**
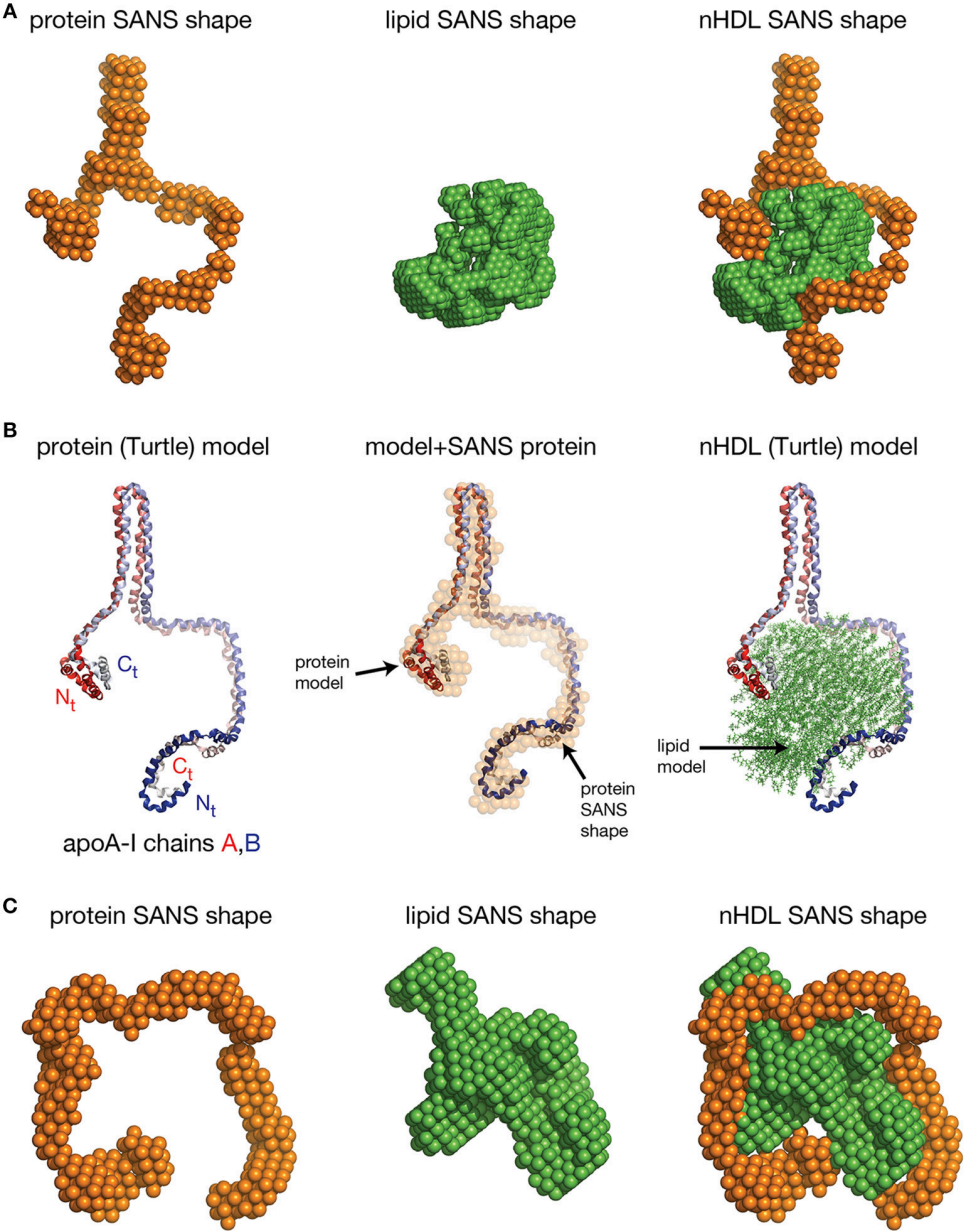
**The low resolution structures nHDL reconstituted with DMPC and with DMPC/FC**. **(A)** Left: The low resolution structure of the protein component of nHDL reconstituted with DMPC. Middle: The low resolution structure of the lipid component of nHDL reconstituted with DMPC. Right: The low resolution structure of nHDL reconstituted with DMPC as a combination of the low resolution structures of the protein and lipid components of nHDL. **(B)** Left: the Y-shaped full length apoA-I dimer in nHDL reconstituted with DMPC proposed by Gogonea et al. (2013). The apoA-I chains shown in cartoon representation and colored with gradient red/blue, are oriented antiparallel in helix 5 registry. Middle: Superposition of the apoA-I double chain with the low resolution structure of the protein component of nHDL reconstituted with DMPC obtained by SANS with contrast variation. Right: The Turtle model of nHDL reconstituted with DMPC; the lipid model was built from 160 DMPC molecules in a combined micellar/lamellar phase. The majority of lipids follow a lamellar arrangement while few of them are arranged radially (as in a micelle) to accommodate the open conformation of apoA-I. **(C)** Left: The low resolution structure of the protein component of nHDL reconstituted with DMPC and FC. The SANS shape resembles a horseshoe. Middle: The low resolution structure of the lipid component of nHDL reconstituted with DMPC and FC. Right: The low resolution structure of nHDL reconstituted with DMPC and FC as a composite of the low resolution structures of the protein and lipid components of nHDL reconstituted with DMPC and FC. The low resolution structures of the protein and lipid components fit each other like a “key in a lock.”

The SANS with contrast variation experiments reviewed so far were performed on nHDL particles reconstituted with full length apoA-I and the more physiologically relevant POPC and FC. The newly discovered open conformation of apoA-I in nHDL raised the following question: is the open conformation of apoA-I a general feature of lipoproteins or is the lipid identity that determines whether the protein adopts an open conformation like in the DSH model, or a ring conformation like in discoidal models? To answer this question, SANS with contrast variation and isotopic labeling of the protein measurements were performed on nHDL particles (9.6 nm) reconstituted with full length apoA-I and DPMC, or DMPC and FC (Gogonea et al., 2013). The low resolution structures of nHDL/DMPC particles (Figure 6A, left) and nHDL/DMPC/FC particles (Figure 6C, left) were found to have open conformation like in nHDL/POPC/FC particles (Figure 5A, left). The authors concluded that the open conformation of apoA-I in nHDL is a general structural feature of these lipoproteins regardless of the type and the amount of lipid used to reconstitute nHDL.

The size of the nHDL particle reconstituted with DMPC measured on native gel (ND-PAGE) was 9.6 nm, but the composition analysis showed that this particle has ~80 DMPC molecules per apoA-I chain (Gogonea et al., 2013). The intriguing “Y-shaped” low resolution structure of apoA-I obtained from SANS with contrast variation suggests that apoA-I in the nHDL/DMPC particle forms a large hairpin (Figure 6A, left) void of lipids (Gogonea et al., 2013). The lipid low resolution structure of this particle has a compact shape (Figure 6A, middle) that fits in between the two arms of the “Y-shaped” low resolution structure of the protein (Figure 6A, right). The open conformation of apoA-I (Figure 6B, left) in the nHDL/DMPC particle implies that, like in nHDL/POPC/FC, the lipid phase is a combination of lamellar and micellar lipid arrangement (Figure 6B, right). The nHDL/DMPC particle exhibits the same distinctive structural features as the nHDL/POPC/FC particle, despite its smaller lipid cargo, that is, the apoA-I double chain has an open conformation (Figure 6B, left) and mixed lamellar-micellar lipid phase (Figure 6B, right, the Turtle model).

The different conformations of apoA-I chains in nHDL/POPC/FC (spiral) and nHDL/DMPC (“Y-shape”) might be puzzling, but these nHDL particles have different lipid compositions and different sizes, so the distinct apoA-I conformations can be attributed to the amount of lipid each particle carries. A spiral conformation of apoA-I can accommodate a larger lipid cargo as it has its entire hydrophobic surface extended and available for interaction with the lipid, while the “Y-shaped” conformation can accommodate a smaller lipid cargo because part of protein hydrophobic surface is void of lipids and remains packed within a hairpin that shields its hydrophobic surface from interaction with water. The thermodynamic stability of the larger or smaller nHDL particle is achieved by balancing interactions between protein and lipids on one hand, and hydrophobic interactions within folded domains of the protein, like hairpins, on the other hand.

Gogonea et al. also reported measurements by SANS with contrast variation and protein isotopic labeling on an nHDL particle reconstituted with DMPC and FC that has a larger lipid phase than the nHDL/DMPC particle (Gogonea et al., 2013). The low resolution structure of the protein in this particle has again an open conformation and resembles a horseshoe (Figure 6C, left); the low resolution structure of the lipid is compact (Figure 6C, middle) and fits within the protein horseshoe shape (Figure 6C, right). The overall shape of the nHDL particle reconstituted with DMPC and FC, given by the combined low resolution structures of the protein and lipid phase, is ellipsoidal (oblate, Figure 6C, right) and comes the closest in overall shape to what discoidal models propose for the structure of nHDL. Still, this nHDL/DMPC/FC particle exhibits the same distinctive characteristics as nHDL/POPC/FC and nHDL/DMPC, that is, the open conformation of apoA-I and the mixed lamellar and micellar organization of the lipid phase.

**Figure 7:**
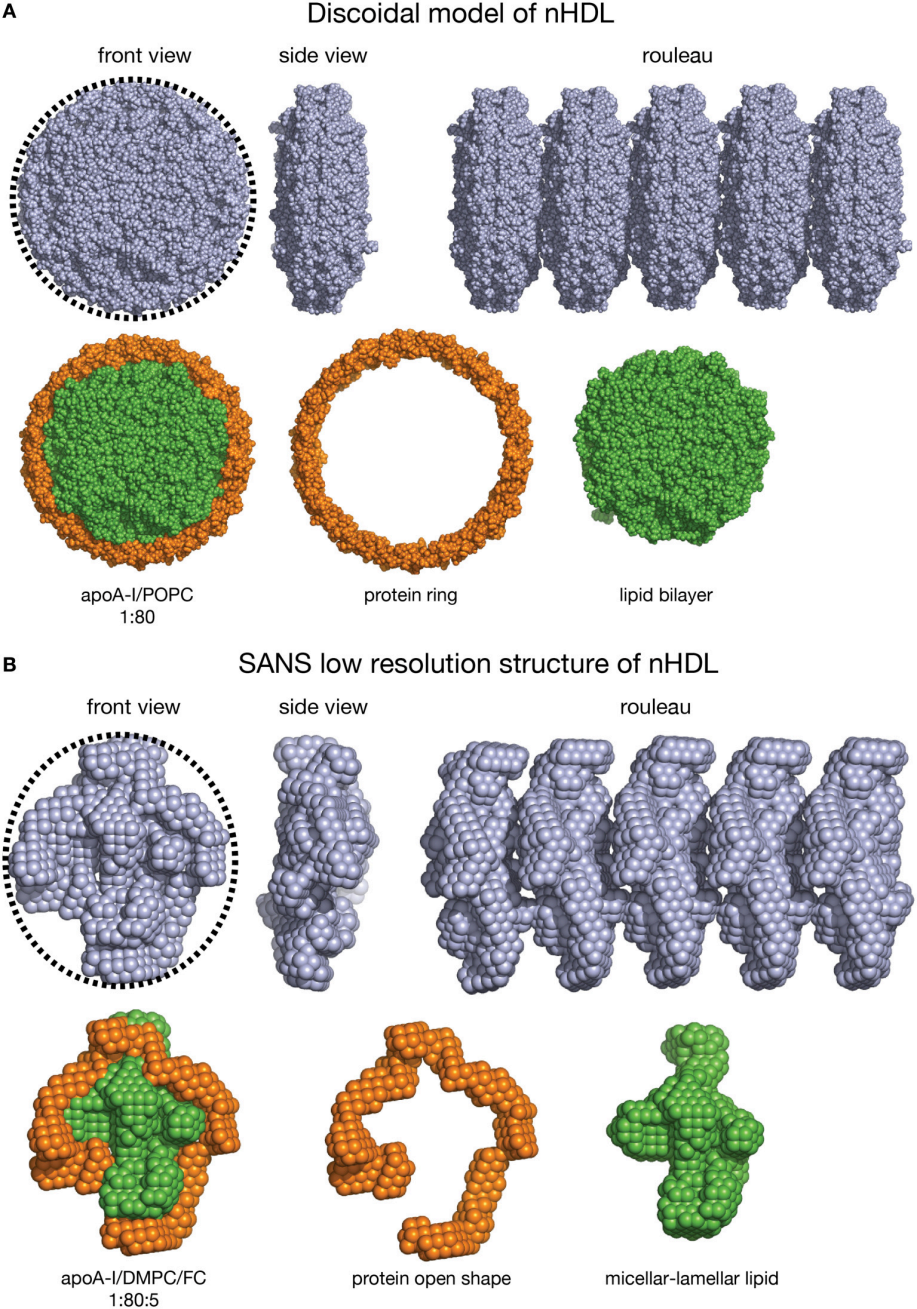
**Shape comparison of the discoidal model and the low resolution structure of nHDL**. **(A)** First *line:* Left: Front view of the discoidal model. A circular dotted black line emphasizes the round shape of the particle. Middle: Side view of the discoidal model; the two views show that the overall shape of the model is a disc. Right: Rouleau formation constructed from several discoidal model shapes stacked as coins. Second *line:* Left: Front view of the discoidal model in which the protein is colored orange and the lipid is colored green. Middle: The protein component of the discoidal model is a ring. Right: The lipid phase of the discoidal model is a bilayer disc. **(B)** First *line:* Left: Front view of the low resolution structure of nHDL/DMPC/FC. A circular dotted black line emphasizes the overall round shape. Middle: Side view of the low resolution structure of nHDL/DMPC/FC; the two views show that the overall shape of the particle is a disc. Right: Rouleau formation constructed from several low resolution shapes stacked as coins. Second *line:* Left: Front view of the low resolution structure of nHDL/DMPC/FC in which the protein is colored orange and the lipid is colored green. Middle: The protein component of nHDL/DMPC/FC, located at the periphery of the lipid, has an open conformation. Right: The lipid phase of the nHDL/DMPC/FC is not a disc. The lipid is a mixture of lamellar and micellar domains.

The nHDL/DMPC/FC particle is similar in lipid composition to the double belt model of nHDL (diameter = 10 nm). Each particle contains 160 PL, thus it can be expected that the overall shape of the nHDL/DMPC/FC particle, as visualized by techniques like EM/cryo-EM/AFM, to be oblate as a discoidal model (Figure 7). On the other hand, these latter visualization methods cannot distinguish between the protein and the lipid components of nHDL, so they cannot tell the exact conformation of the protein in the particle. The shapes of the discoidal model and the low resolution structure of nHDL/DMPC/FC are shown in Figure 7 as they would appear in an EM/cryo-EM/AFM image. Both front view images have round shapes (emphasized by the circular dotted line circumscribing them, Figures 7A,B, left), but the protein shape cannot be distinguished within the particle, i.e., one cannot say whether the protein is a ring or has an open shape. In addition, Figure 7 shows a hypothetical rouleau formation (Figures 7A,B right), which was used in many EM studies as a “proof/confirmation” that nHDL particles are dicoidal and stack like coins. The rouleau constructs built with the model (Figure 7A right) and the low resolution (Figure 7B right) shapes look similar. When the protein and the lipid are “painted” differently (Figures 7A,B, second line: protein = orange, lipid = green) as the SANS with contrast variation would do, then the protein conformation within the particle can be visualized (Figure 7B, second line). Note that SANS with contrast variation “paints” individual components of the lipoprotein by using different concentrations of D_2_O in the buffer (*vide supra*). An equivalent technique does not exist for EM/cryo-EM/AFM methods.

Still, it is worth noting that a recent cryo-EM study produced a “visualization” of the protein component of nHDL by combining many individual cryo-EM images and processing them through computer algorithms (Zhang et al., 2011; Zhang and Ren, 2012). While the authors of this study concluded that their cryo-EM images are consistent with the discoidal model of nHDL, their Figure 5 shows that apoA-I is disjoined (not a ring like in discoidal models). Their image of the protein is similar to the SANS low resolution structure of nHDL/DMPC/FC. Nevertheless, while the SANS low resolution structure is a direct experimental observation, the electron density of the protein component of apoA-I is “extracted” from the electron density of the entire particle, as seen in the cryo-EM images, by computer algorithms (Figure 5 in Zhang and Ren, 2012), and thus does not correspond to its visualization as an individual entity like in SANS with contrast variation.

#### Folding dynamics and plasticity of amphipathic apoA-I is the basis for nHDL discreet size variation

One of apoA-I's most important structural features is its plasticity, i.e., its ability to “shrink” or “extend” and bind lipid cargos in discreet sizes. The feature is critical for the formation of nHDL and the continuous remodeling of the lipoprotein during its lifetime as it progresses along the RCT pathway. While the feature was early recognized, the detailed molecular mechanism by which apoA-I transits from one HDL size to another is still debated (Brouillette and Anantharamaiah, 1995; Li et al., 2004; Gogonea et al., 2013).

In the mid-nineties, when the picket fence model of nHDL grew in acceptance, it was suggested that apoA-I transits from one HDL discreet size to the next by adjusting the number of 11 or 22 amphipathic repeats in the “fence” it makes at the periphery of the lipid phase (Brouillette and Anantharamaiah, 1995). When the lipid core is small, the repeats left out of the “fence” self-associate in smaller bundles to mutually shield their hydrophobic surface from exposure to water. This mechanism is supported by the observation that two amphipathic repeats are required for fence extension to incrementally increase the size of nHDL (Brouillette et al., 1984; Jonas et al., 1989).

At the same time, immuno-reactivity investigations of apoA-I conformational changes in nHDL of various sizes with variable cholesterol content pointed to a central region of apoA-I, P_99_–P_143_, which responds to lipid phase enlargement by significantly altering antibody binding to the N_t_ and the central region, i.e., the expansion of the PL phase strengthens antibody binding competition to adjacent apoA-I epitopes. This result was understood as evidence that lipids bind to apoA-I domain P_99_–P_121_ in larger nHDL particles (Collet et al., 1991; Marcel et al., 1991; Calabresi et al., 1993; Bergeron et al., 1995). On the other hand, the increase in cholesterol content changes the antibody binding capacity to only two central epitopes, P_99_–Q_132_ and K_118_–M_148_, and the N_t_ epitope E_2_–W_8_. This effect of cholesterol presence on apoA-I central region was later confirmed by SANS with contrast variation experiments, which showed that the large hairpin formed in the central region of apoA-I in nHDL/DMPC (Figure 6A, left) opens up when cholesterol is present (Figure 6C, left; Gogonea et al., 2013).

A decade later, when the double belt model replaced the picket fence model as the preferred way to describe the structure of nHDL, the mechanism for HDL incremental expansion was redefined by taking into consideration that the N_t_ mutant of apoA-I (Δ43–apoA-I) was the structural basis for the double belt model. Li et al. proposed that nHDL particles larger than 9.6 nm form when the apoA-I double chain ring expands by incorporating segments from the lipid-free N_t_ (Li et al., 2004), while HDL particles smaller than 9.6 nm form when the apoA-I ring contorts into an out of plane conformation, still binding to lipid along its entire perimeter (i.e., helices h1–h10; Gu et al., 2010).

The observation of the low resolution structure of nHDL/DMPC obtained from SANS with contrast variation (Figure 6A, left; Gogonea et al., 2013) invited a reassertion of the nHDL expansion mechanism. The fact that the Turtle model of nHDL (Figure 6A, right) contains a large hairpin formed by lipid free amphipathic repeats of apoA-I argues that full length apoA-I in a 9.6 nm HDL particle do not bind PLs along its entire perimeter, unless the lipid cargo is large enough (like in the DSH model). The Turtle model implies that both the N_t_ and C_t_ are bound to PL while the middle region of apoA-I is lipid free, in contrast to the model proposed by Li et al., in which N_t_ is lipid free, while the central region and C_t_ of apoA-I bind to lipid (Li et al., 2004).

The revisited nHDL expansion mechanism (Gogonea et al., 2013) is similar with the one suggested earlier for the picket fence model (Brouillette et al., 1984; Jonas et al., 1989) and the immuno-reactivity studies (Collet et al., 1991; Marcel et al., 1991; Calabresi et al., 1993; Bergeron et al., 1995), in the sense that it assumes that the N_t_ and C_t_ of apoA-I bind to PL in nHDL particles with smaller lipid cargos, while the central region of apoA-I binds PL when the particle expands through a growing lipid phase.

In conclusion, earlier biophysical (i.e., attenuated total reflection IR spectroscopy, (Wald et al., 1990b); ^13^C NMR, (Sparks et al., 1992a); CD) and theoretical studies (Brasseur et al., 1990) concluded that apoA-I conformation is different in nHDL of different sizes, containing two A–I chains (Brouillette and Anantharamaiah, 1995), an assumption reaffirmed by the latest SANS with contrast variation experiments (Wu et al., 2009; Gogonea et al., 2013).

## Models of spherical HDL

The second step in RCT is nHDL maturation, the conversion of nHDL particles into spherical HDL (sHDL) by plasma enzyme lecithin cholesteryl acyltransferase (LCAT) that transforms FC into cholesterol ester (CE) on the surface of nHDL (Zannis et al., 2006). Cholesterol ester moves from particle's surface into its core, and triglycerides (TG) join the sHDL lipid core through lipid transfer mediated by other plasma enzymes (CETP) and cell receptors (SR-BI; Zannis et al., 2006). As the lipid core grows it pushes back PL partially exposing their acyl chains to solvent and thermodynamically destabilizing the HDL particle, which recruits additional apoA-I molecules to reduce its exposed hydrophobic surface (Brouillette and Anantharamaiah, 1995).

In plasma, the pool of sHDL particles is heterogeneous and contains particles of different sizes (8.8–11 nm) and with various protein/lipid compositions (Huang et al., 2011). The protein component of sHDL contains two to seven apoA-I chains (Huang et al., 2011), while the lipid phase packs variable amounts of CE and TG with the number of PL greatly diminished compared to nHDL (20–30 PL per apoA-I chain; Wu et al., 2011).

### Conceptual and MD simulation models of sHDL

As with nHDL, the lack of a crystal structure for sHDL lead researchers to rely on alternative interrogation techniques into the structure of sHDL [CD, MS-crosslinking (Silva et al., 2008), HDX-MS (Chetty et al., 2013), SANS (Wu et al., 2011), EM (Zhang et al., 2011)]. Unlike nHDL, homogeneous sHDL particles are more difficult to obtain by reconstitution. The procedure to prepare sHDL particles involves mixing nHDL with LCAT and low density lipoprotein (LDL; Jonas et al., 1990). Inspired by the crystal structure of the truncated lipid free apoA-I (Δ43–apoA-I), Borhani et al. were among the first to propose a model for an sHDL particle with four apoA-I chains, in which apoA-I is organized as two intersecting double belts (Borhani et al., 1997). Silva et al. investigated the structure of sHDL particles reconstituted with two and three apoA-I chains (Jonas et al., 1990) by employing MS-crosslinking (Silva et al., 2008), and discovered that all crosslinks found in nHDL are preserved in sHDL. The authors concluded that the arrangement of the three apoA-I chains in sHDL mimics the apoA-I arrangement in nHDL to some extent, that is, the protein chains are oriented antiparallel with respect to each other in helix 5 registry. To preserve the inter-chain interaction surface between the two apoA-I chains present in the double belt model (Segrest et al., 1999), the authors proposed that the three apoA-I chains be paired with each other for half of their length to form a symmetric construct made of three folded rings coined the Trefoil model (Silva et al., 2008). This model employs N_t_ truncated apoA-I (Δ43–apoA-I) and does not include a model for the lipid phase (Silva et al., 2008). Huang et al. extended the Trefoil concept to sHDL particles with more than three apoA-I chains (Huang et al., 2011).

At about the same time Segrest et al. proposed a MD simulation model of an sHDL particle containing two truncated apoA-I chains (Δ43–apoA-I). The authors performed shorter all atom force field MD simulations and longer simulations by using a coarse-grained force field for a simplified description of the lipids and protein molecules. The simulation (NPT ensemble) was performed at 310 K after a temperature jump at 410 K (Catte et al., 2008). The authors concluded that the protein component is largely rigid and cholesteryl ester (CE) molecules contribute to protein's low mobility. In a second MD simulation study on sHDL Segrest et al. investigated sHDL particles containing two or three apoA-I chains and lipid compositions similar to those found in circulating sHDL (Segrest et al., 2013). The simulation was carried out at 310 K and 1 atm. Spherical HDL particles with specific composition were assembled and subjected to 5 and 10 ns simulation, and then to 30 ns of MD-simulated annealing. The authors found that reconstituted sHDL particles contain an excess of phospholipids as compared to circulating sHDL particles, and concluded that models of reconstituted sHDL are less relevant for describing the properties of circulating sHDL.

Chetty et al. (2013) carried out an HDX-MS study of sHDL isolated from plasma, and confirmed that apoA-I secondary structure and overall dynamics/stability in sHDL are similar to what was found in nHDL (Chetty et al., 2012). In addition, Chetty et al. identified the domain 115–158 of apoA-I (helix 5) in sHDL as very dynamic (displays bimodal HDX kinetics) and exhibiting a helix-to-loop transition on a time scale of minutes, as in nHDL (Chetty et al., 2012, 2013).

### The low resolution structure of sHDL

**Figure 8:**
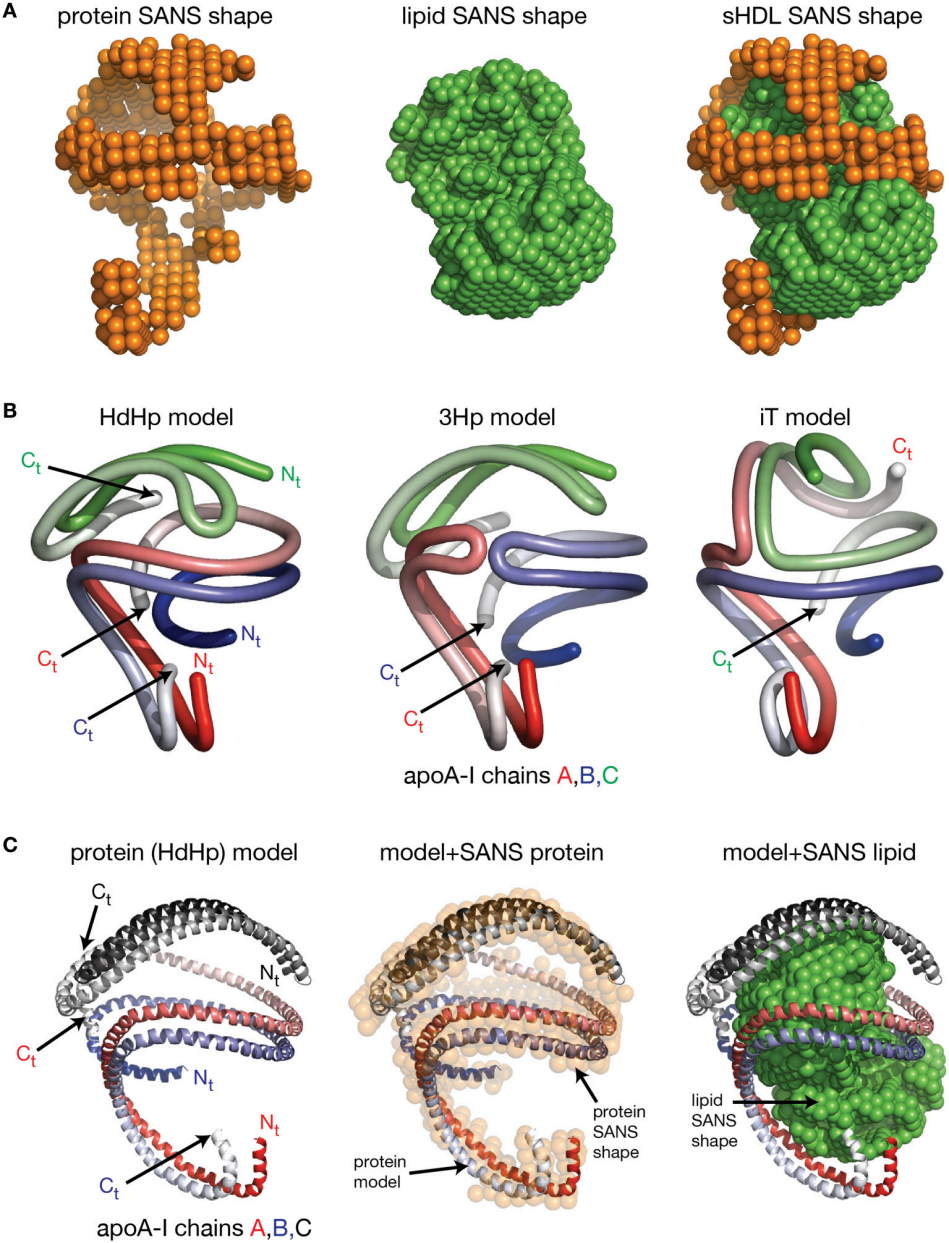
**The low resolution structure and models of sHDL**. **(A)** Left: The low resolution structure of the protein component of sHDL obtained from SANS with contrast variation. Middle: The low resolution structure of the lipid component of sHDL. Right: The low resolution structure of sHDL as a composite of the low resolution structures of the protein and lipid components of sHDL. **(B)** Schematic representation of the three apoA-I chains in three hypothetical models of sHDL proposed by Wu et al. (2011): HdHp, 3Hp, and iT. Tubes colored with gradient red, blue and green are used to represent the protein chains. Left: The HdHp model of sHDL. Middle: The 3Hp model of sHDL; the locations of C_t_ of chains A and B are interchanged with respect to the HdHp model. Right: The iT model of sHDL; the locations of C_t_ of chains A and C are interchanged with respect to the HdHp model. **(C)** Left: The HdHp model of the apoA-I trimer in sHDL. The apoA-I chains shown in cartoon representation are colored with gradient red/blue/gray. The chains in the AB dimer are oriented antiparallel in helix 5 registry. The chain C is folded into a hairpin and interacts for the AB dimer in specific regions of apoA-I. Right: Superposition of the protein model (HdHp) and the lipid low resolution structure. The authors have not proposed a model for the lipid component of sHDL.

Low resolution structures of the protein and lipid components of sHDL reconstituted with three apoA-I chains (Jonas et al., 1990), obtained from SANS with contrast variation and isotopic labeling of the protein, were recently published by Wu et al. (2011), and are shown in Figure 8A (left and middle panels, respectively), and the low resolution structure of the sHDL particle is shown in Figure 8A, right panel, as a composite of the low resolution structures of the protein and lipid components of sHDL. Wu et al. investigated three possible architectures of the three apoA-I chains that can fit within the protein low resolution structure: the Helical-dimer/Hairpin model (HdHp), the 3 Hairpins model (3Hp), and the integrated Trimer model (iT) (Wu et al., 2011).

In the HdHp model (Figure 8B, left) the three apoA-I chains are organized as a combination of an apoA-I dimer with the chains oriented antiparallel in helix 5 registry as found in nHDL, and an apoA-I monomer in a hairpin conformation. The apoA-I dimer is rather contorted with a spiral shape reminiscent of the DSH model of nHDL, and the helical dimer and the hairpin monomer interact with each other at specific locations along the apoA-I chain (Wu et al., 2011). In the 3Hp model (Figure 8B, middle) three apoA-I monomers in contorted hairpin conformation are arranged to fit the protein low resolution structure. The iT model (Figure 8B, right) resembles in spirit to the Trefoil model, but unlike the latter, it has only one node where the three apoA-I chains with contorted shape converge. The iT model is less symmetric than the Trefoil model, uses full length apoA-I chains, and does not contain protein rings.

The three SANS derived models of sHDL (HdHp, 3Hp, iT) fit well the protein low resolution structure (Wu et al., 2011), thus to discriminate among them and find the best candidate for the structure of sHDL, Wu et al. used MS-crosslinking (Wu et al., 2011). The authors found that only the HdHp model (Figure 8C) satisfies all 23 crosslinks reported by Silva et al. (2008) for sHDL and concluded that the protein component of sHDL is a combination of a spiral dimer and a hairpin monomer.

Based on this model of sHDL, Wu et al. suggested that a thermodynamically stable sHDL forms during maturation when an apoA-I monomer in plasma joins a thermodynamically destabilized sHDL particle with a growing CE core and increased lipid hydrophobic surface exposed to solvent. This contrast with the significant entropy barrier one would anticipate forming a trefoil configuration from a double belt apoA-I and an apoA-I monomer because the apoA-I dimer requires unzipping halfway as the third apoA-I chain simultaneously anneals with the unzipped portion to form the two nodes where the three apoA-I chains join in the Trefoil model.

**Figure 9:**
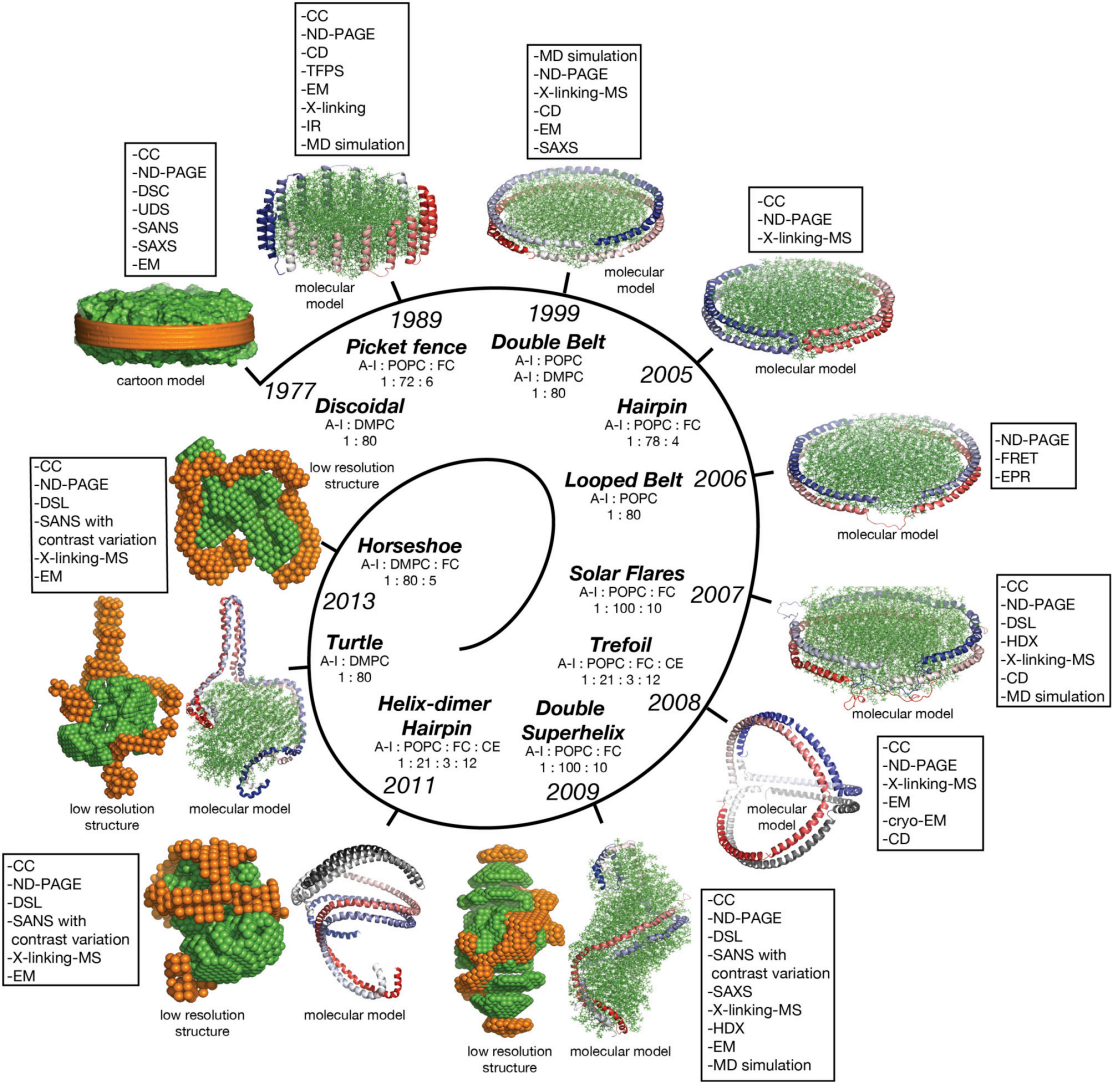
**Historical perspective of the development of various nHDL and sHDL models**. The map displays in chronological order various models of nHDL and sHDL developed in the last four decades. Alongside each model are listed various biophysical methods used to develop a particular model. While some of the models were not initially developed by incorporating data from all experimental techniques listed, overtime, some of the listed techniques were used to support that particular model. The low resolution structures obtained from SANS with contrast variation are displayed next to the nHDL and sHDL models derived from them. Legend: A-I, apolipoprotein A-I; CC, chemical composition analysis; CD, circular dichroism; cryo-EM, cryo electron microscopy; DMPC, dimyristoyl-phosphatidylcholine; DSC, differential scanning calorimetry; DSL, dynamic light scattering; CE, cholesteryl ester; EM, negative staining electron microscopy; EPR, electron paramagnetic resonance spectroscopy; FC, free cholesterol; FRET, Förster resonance energy transfer; IR, infrared spectroscopy; ND-PAGE, non-denaturing PAGE gel electrophoresis; POPC, palmitoyl-oleoyl-phosphatidylcholine; SANS, small angle neutron scattering; SAXS, small angle X-ray scattering; TFPS, tryptophan fluorescence polarization spectra; UDS, ultraviolet difference spectroscopy; X-linking-MS, crosslinking mass spectrometry.

A chronological perspective of the development of nascent and spherical HDL models is given in Figure 9. The figure lists for each model the particle composition and various experimental techniques used to derive or confirm the model.

## Changes in apoA-I structure alter HDL functionality

Numerous structural and functional studies of HDL role in reverse cholesterol transport (RCT) demonstrated that apoA-I is not only a scaffold for lipids that gives structural integrity to HDL particles, but it also provides a functional interaction interface for various plasma enzymes [e.g., lecithin cholesteryl acyltransferase (LCAT), myeloperoxidase (MPO), paraoxonase 1 (PON1), cholesteryl ester transfer protein (CETP)] and cell receptors (e.g., the ABCA1, ABCG1, and SR-BI) encountered along the RCT pathway. In other words, cell receptors and plasma enzymes use various epitopes of apoA-I to bind HDL and modify its lipid cargo.

Amino acid substitutions and deletions were extensively used to enrich our knowledge of apoA-I conformation and to better understand structural features of particular domains of the protein (Bruhn and Stoffel, 1991; Minnich et al., 1992; Sorci-Thomas et al., 1993; Ji and Jonas, 1995; Schmidt et al., 1995; Sorci-Thomas and Thomas, 2002). Many naturally occurring and bioengineered mutations (Table 2; Brouillette and Anantharamaiah, 1995; Sorci-Thomas and Thomas, 2002) and posttranslational modifications (Smith, 2010; Rosenson et al., 2016) in apoA-I have been reported and shown to alter HDL function or make it dysfunctional (e.g., by chlorination, oxidation, or nitration of specific apoA-I residues), resulting in impaired cholesterol efflux, change in HDL phenotype, and increased risk of coronary artery disease (CAD; Rosenson et al., 2016). Therefore, new diagnostic and therapeutic tactics to treat cardiovascular disease (CVD) might emerge from a better understanding of the structural aspects of dysfunctional HDL.

**Table 2:** **Compilation of naturally occurring and bioengineered mutations in human apoA-I^a^**.

**Mutation**	**Effect on HDL structure and function**	**Mutation**	**Effect on HDL structure and function**
P_3_R, von Eckardstein et al., 1989	Interferes with the formation of a β-turn in N_t_	R_149_A, Koukos et al., 2007b	Reduces LCAT activity
P_3_H, von Eckardstein et al., 1989	(apoA-I_Munster3C_) interferes with the formation of a β-turn in N_t_	R_149_V, Sviridov et al., 2000	Does not affect α-helical structure, reduces LCAT activity
P_4_R, von Eckardstein et al., 1989	(apoA-I_Munster3B_) has no known effect on RCT	*R*_151_*C*, Daum et al., 1999a	(apoA-I_Paris_) inhibits LCAT activation, reduces HDL levels
R_10_L, Ladias et al., 1990	(apoA-I_Baltimore_) has no known effect on HDL phenotype	*R*_153_*P*, Esperoìn et al., 2008	(apoA-I_Montevideo_) lowers HDL levels and is associated with CAD
D_13_Y, Takada et al., 1991	(apoA-I_Yame_) has no known effect on HDL phenotype	*V*_156_*E*, Huang et al., 1998	(apoA-I_Oita_) decreases the levels of HDL in plasma and inhibits LCAT activation
*Q_17_P+FS*^c^*X_26_*, Pisciotta et al., 2008	Frame shift (FS) and stop codon (X) lowers HDL plasma levels	*A*_158_*E*, Mahley et al., 1984	(apoA-I_Munster2B_) is associated with low levels of HDL in plasma
G_26_R, Nichols et al., 1988	Initiates deposition of mutant proteins or proteolytic cleaved fragments	*L*_159_*R*, Miettinen et al., 1997	(apoA-I_Fin_) is associated with low levels of HDL in plasma
A_37_T, Matsunaga et al., 1991	Does not create apoA-I deficiency	*L*_159_*P*, Miller et al., 1998	(apoA-I_Zavalla_) is associated with low levels of HDL in plasma and premature CAD
W_50_R, Booth et al., 1995	Causes hereditary amyloidosis	*R*_160_*L*, Daum et al., 1999b	(apoA-I_Oslo_) is associated with low levels of HDL in plasma and LCAT inhibition
S_52_C, Zhu et al., 2005	Has no known effect on HDL phenotype	H_162_Q, Moriyama et al., 1996a; Hoang et al., 2003	(apoA-I_Kurume_) does not produce accelerated atherosclerosis, reduces LCAT activity
L_60_R, Soutar et al., 1992	Causes autosomal dominant amyloidosis	*H*_162_–*A*_207_^c^; Δ*K*_208_–*Q*_243_, Moriyama et al., 1996b	Leads to low apoA-I and HDL levels and bilateral xanthomas of the Achilles tendon, elbow, knee joint, and corneal opacities
ΔL_60_–F_71_^b^; ins V,T, Booth et al., 1996	Causes hereditary hepatic and systemic amyloidosis	*P*_165_*R*, von Eckardstein et al., 1989	is associated with lower apoA-I and HDL levels
ΔE_70_–W_72_, Persey et al., 1998	Causes hereditary nephropathic systemic amyloidosis	Δ*P*_165_–*A*_175_, Martin-Campos et al., 2002	(ApoA-I_Mallorca_) impairs LCAT activation and induces dominant familial hypoalphalipoproteinemia
N_74_C, Zhu et al., 2005	Slightly lowers cholesterol efflux	Y_166_F, Wu et al., 2007	Impairs LCAT activity
D_89_E, von Eckardstein et al., 1990	Mutation in less conserved domain of apoA-I, has no known effect on RCT	E_169_Q, von Eckardstein et al., 1990	Effect on HDL phenotype is unknown
L_90_P, Asl et al., 1999a	Causes hereditary amyloid cardiopathy	R_173_P, Asl et al., 1999b	Is associated with cardiac and cutaneous amyloidosis
A_95_D, Araki et al., 1994	(apoA-I_Hita_) does not affect HDL phenotype	*R*_173_*C*, Weisgraber et al., 1980	(apoA-I_Milano_) is associated with reduced plasma levels of HDL and elevated TG levels
Y_100_H, Moriyama et al., 1996a	Does not accelerate atherosclerosis	R_177_H, Assmann et al., 1993	Effect on HDL phenotype is unknown
D_103_N, Menzel et al., 1982	(apoA-I_Munster3A_) effect on HDL phenotype is not known	L_178_H, Petrlova et al., 2012	Leads to altered conformation, decreased stability, reduced lipid binding capacity, forms fibrils
ΔK_107_, Amarzguioui et al., 1998	(apoA-I_Munster2A_) leads to extensive intimal amyloid deposits	*L*_178_*P*, Hovingh et al., 2004	Lowers HDL levels, leads to endothelial dysfunction, increased arterial wall thickness, CAD
Δ*K*_107_, Rall et al., 1984	(apoA-I_Marburg_) inhibits LCAT activation	Δ*E*_191_–*P*_220_, Nagao et al., 2014	Is associated with defective lipid binding and lower HDL levels
K_107_C, Zhu et al., 2005	Increases cholesterol efflux	*K*_195_*C*, Zhu et al., 2005	Reduces lipid binding capability and impaires cholesterol efflux
K_107_M, von Eckardstein et al., 1990	Effect on HDL phenotype not known	E_198_K, Assmann et al., 1993	(apoA-I_Munster4_) effect on HDL phenotype is unknown
W_108_R, Araki et al., 1994	(apoA-I_Tsushima_) does not affect HDL phenotype	*L*_203_–*F*_229_^c^; Δ*L*_230_–*Q*_243_, Funke et al., 1991	Causes HDL deficiency, partial LCAT inhibition, and corneal opacity
E_110_K, Takada et al., 1990; Hoang et al., 2003	(apoA-I_Fukuoka_) does not affect HDL phenotype, reduces LCAT activity	D_213_G, Mahley et al., 1984	(apoA-I_Munster3D_) effect on HDL phenotype is not know
*G*_129_*C*, Zhu et al., 2005	Increases structural stability, reduces lipid binding capability, slightly lowers cholesterol efflux	*L*_218_*A*, Fotakis et al., 2013b	Is associated with decrease in plasma cholesterol and apoA-I levels
E_136_K, Mahley et al., 1984	(apoA-I_Norway_) effect on HDL phenotype is not known	*L*_219_*A*, Fotakis et al., 2013b	Is associated with decrease in plasma cholesterol and apoA-I levels
*E*_136_*X*, Dastani et al., 2006	Produces circulating HDL deficiency	*V*_221_*A*, Fotakis et al., 2013b	Is associated with decrease in plasma cholesterol and apoA-I levels
E_139_G, Assmann et al., 1993	Effect on HDL phenotype is not known	*L*_222_*A*, Fotakis et al., 2013b	Is associated with decrease in plasma cholesterol and apoA-I levels
ΔR_140_–D_150_, Sviridov et al., 2000	Affects α-helical structure, reduces LCAT activation	*E*_223_*A*, Fotakis et al., 2013b	Is associated with alterations in HDL phenotype
R_140_–D_150_ 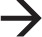 Q_63_–D_73_, Sviridov et al., 2000	Does not affect α-helical structure, reduces LCAT activation	Δ*E*_223_–*Q*_243_, Fotakis et al., 2013b	Leads to critical loss of lipid binding and low HDL levels
*L*_141_*R*, Miccoli et al., 1997	(apoA-I_Pisa_) influences efflux of cholesterol into plasma and interferes with the formation of HDL	*F*_225_*A*, Fotakis et al., 2013a	Is associated with decrease in plasma cholesterol, HDL, and apoA-I levels
P_143_R, Assmann et al., 1993	(apoA-I_Giessen_) effect on HDL phenotype is not known	*K*_226_*A*, Fotakis et al., 2013b	Is associated with alterations in HDL phenotype
P_143_A, Sviridov et al., 2000	Affects α-helical structure, reduces LCAT activation	*V*_227_*A*, Fotakis et al., 2013a	Decreases plasma cholesterol, HDL and apoA-I levels
*L*_144_*R*, Recalde et al., 2001	apoA-I_Zaragoza_ decreases the levels of HDL in plasma, HDL has more TG and less CE	*F*_229_*A*, Fotakis et al., 2013a	Decreases plasma cholesterol, HDL and apoA-I levels
*L*_144_*P*, Recalde et al., 1998	Decreases the levels of HDL in plasma, has no effect on CVD	*L*_230_*A*, Fotakis et al., 2013a	Decreases plasma cholesterol, HDL and apoA-I levels
Δ*E*_146_–*R*_160_, Deeb et al., 1991	Leads to plasma apoA-I and HDL cholesterol below 15% of normal levels	Δ*E*_235_, Han et al., 1999	(apoA-I_Nichinan_) is associated with decreased protein stability and low plasma HDL levels
E_147_V, von Eckardstein et al., 1990	Effect on HDL phenotype is not known		

a *ApoA-I mutations associated with hereditary amyloidosis (underline font), mutations associated with low HDL plasma levels and LCAT deficiency (italic font) and mutations with unknown effect (normal font)*.

b *Deletion*.

c *Frame shift*.

### Naturally occurring and bioengineered apoA-I mutations and their effect on HDL structure and function

More than 75 naturally occurring and bioengineered mutations in human apoA-I were reported (Table 2) to induce structural modifications in lipid free and lipid bound apoA-I and/or to alter the HDL phenotype *in vivo*. Approximately half of mutations decrease HDL plasma levels and can be grouped into amino acid substitutions that inhibit LCAT activity to some extent or are associated with amyloidosis (Sorci-Thomas and Thomas, 2002). The mutations that lead to LCAT inhibition are mainly located within repeats 5, 6, and 7 of apoA-I (P_121_–G_186_; Sorci-Thomas et al., 2009), while those responsible for amyloid deposition are mostly encountered in the N_t_ of apoA-I (D_1_–L_90_). Alteration of HDL function by mutations suggests that changes in the tertiary structure of lipid bound apoA-I impacts plasma levels of HDL.

#### Amino acid substitutions associated with hereditary amyloidosis

In hereditary amyloidosis insoluble amyloid fibrils form when apoA-I mutants, like apoA-I_Iowa_ (G_26_R; Nichols et al., 1988; Das et al., 2014) or W_50_R (Booth et al., 1995; Das et al., 2014) aggregate, or when proteolytic cleaved fragments (D_1_–R_83_) progressively accumulate, leading to organ failure and death. Mutants of apoA-I associated with hereditary amyloidosis and low HDL plasma levels are listed in Table 2 with underlined font.

#### Amino acid substitutions that inactivate LCAT

Naturally occurring amino acid substitutions in human apoA-I associated with low HDL levels and diminished LCAT catalytic efficiency have been reviewed extensively (Sorci-Thomas and Thomas, 2002; Kunnen and van Eck, 2012; Schaefer et al., 2014; Saeedi et al., 2015). Numerous mutation/deletion studies identified the apoA-I domains that are critical for LCAT interaction with HDL (Cho et al., 2001; Sorci-Thomas and Thomas, 2002; Alexander et al., 2005; Koukos et al., 2007a; Boes et al., 2009; Sorci-Thomas et al., 2009; Roshan et al., 2011; Fotakis et al., 2013a, 2015). A large body of experimental evidence suggests that structural modifications within repeats 5, 6, and 7 of apoA-I (P_121_–G_186_) alter its conformation preventing LCAT from binding to HDL or reducing its catalytic efficiency in converting FC to CE, leading to a decrease in plasma levels of mature HDL and a disruption in the RCT cycle (Dhoest et al., 1997; Frank et al., 1998; Sorci-Thomas et al., 1998, 2009; Sviridov et al., 2000; Roosbeek et al., 2001).

For example, HDL reconstituted with apoA-I_Oita_ (V_156_E) is similar in size and composition to HDL reconstituted with wild-type apoA-I, but displays significantly reduced LCAT catalytic efficiency (Cho and Jonas, 2000). On the other hand, apoA-I_Seattle_, which lacks residues E_146_–R_160_ from repeat 6, (P_143_–A_164_), forms small HDL particles (7–8 nm) and was linked to bilateral arcus senilis (Deeb et al., 1991). Analysis of plasma samples containing ApoA-I_Mallorca_ mutant lacking residues P_165_–A_175_ from repeat 7 (P_165_–G_186_), revealed lower HDL levels, increased triglycerides (TG), and decreased CE content (Martin-Campos et al., 2002). Another mutant, apoA-I_Fin_ features a positive charge motif (R_159_–R_160_) as a result of substituting a leucine to an arginine (L_159_R); this mutation leads to lower HDL plasma levels and makes the protein susceptible to hypercatabolism. In apoA-I_Zavalla_ (L_159_P) the same residue (L_159_) is replaced by proline making the mutant less susceptible to proteolytic cleavage (no fragments detected *in vivo*), but small unstable HDL particles are produced, presumably due to the extra kink that the additional proline creates. However, none of these mutants (apoA-I_Fin_ and apoA-I_Zavalla_) were associated with CAD (Miettinen et al., 1997).

ApoA-I_Milano_ has an arginine substituted with a cysteine (R_173_C) within repeat 7 (P_165_–G_186_; Weisgraber et al., 1980), which allows apoA-I dimers to covalently link through disulfide bonds. The large proportion of HDL particles having apoA-I chains locked through a disulfide bond contributes to the observed restricted HDL size, lower HDL plasma levels, and impaired LCAT activity reported for apoA-I_Milano_(Calabresi et al., 1994; Sirtori et al., 2001; Nissen et al., 2003; Bhat et al., 2010). These effects on HDL phenotype should increase the risk for atherosclerosis (Sirtori et al., 2001), but actually atherosclerotic lesions in patients with CAD regressed when apoA-I_Milano_ was administered intravenously (Nissen et al., 2003). Because the apoA-I_Milano_ chains align in helix 7 registry rather than helix 5 registry (as wild type apoA-I does), it was suggested that apoA-I_Milano_ adopts a different conformation in HDL than regular apoA-I does (Calabresi et al., 1994), however, MD simulations concluded that apoA-I_Milano_ dimers retain the double belt conformation of wild type apoA-I in nHDL (Klon et al., 2000) except for N_t_ and C_t_ that buckle around the lipid instead of folding back to the protein central domain (Bhat et al., 2010). Similarly to apoA-I_Milano_, apoA-I_Paris_ (R_151_C, in repeat 5, P_143_–A_164_) forms homodimers that restrict apoA-I mobility and probably alters its conformation, nonetheless this mutant does not seem to alter HDL phenotype (Daum et al., 1999a). Finally, in a study employing an apoA-I double mutant, charged amino acids were substituted with non-polar ones (R_160_V/H_162_A) leading to loss in LCAT catalytic activity similar to single mutations R_160_L and H_162_Q, however the double mutant protein has not acquired a significant change in secondary structure or lipid binding properties as the single mutants (Gorshkova et al., 2006).

#### Other mutations in apoA-I

Several mutations in apoA-I C_t_ were investigated because this domain of apoA-I plays a key role in the initial steps of apoA-I lipidation (Chroni et al., 2003; Kono et al., 2010; Fotakis et al., 2013a,b; Nagao et al., 2014). For example, Fotakis et al. showed that apoA-I mutants F225A/V227A/F229A/L230A inhibit HDL formation in mice (Fotakis et al., 2013b), while Kono et al. reported that apoA-I_Nichinan_ mutant (single amino acid deletion, ΔE_235_) has reduced lipid clearance capability (Kono et al., 2010). In another study, Jayaraman et al. showed that an apoA-I mutant, in which all four tryptophan residues are replaced by phenylalanine, 4WF, significantly reduces ABCA1 mediated cholesterol efflux (Jayaraman et al., 2011a). Chroni et al. reported a similar effect following the deletion of the P_220_–S_231_ region, while no change in HDL phenotype was observed when A_232_–Q_243_ domain was deleted (Chroni et al., 2003).

### Effects of apoA-I posttranslational modifications on RCT

Posttranslational modifications (PTM) of apoA-I (chlorination, oxidation, nitration, carbamylation, etc.) make HDL dysfunctional, and there is a large and growing body of clinical and functional studies suggesting that dysfunctional HDL is directly associated with atherosclerosis and CVD progression (Fisher et al., 2012). For example, Shao et al. showed that the modification of apoA-I lysines by aldehydes (acrolein and malondialdehyde) inhibits cholesterol efflux mediated by ABCA1 (Shao et al., 2005a, 2010). On the other hand, several research labs identified MPO as a major enzymatic factor that chemically modifies apoA-I into various oxidized forms (e.g., chloro-, bromo-, oxy-, nitro-, nitrile-, carbamyl-, etc.) that are abundant in atherosclerotic lesions and detectable in plasma of CAD patients (Heinecke, 2003; Pennathur et al., 2004; Zheng et al., 2004; Nicholls et al., 2005; Undurti et al., 2009; Shao et al., 2012). For example, site-specific chlorination of tyrosine (Peng et al., 2005; Shao et al., 2005b; Zheng et al., 2005; Wu et al., 2007; DiDonato et al., 2014) and site-specific tryptophan oxidation (Trp${}_{72}^{\text{Ox}}$-apoA-I, Huang et al., 2014) are instances of apoA-I PTMs that impair ABCA1-mediated cholesterol efflux and reduce LCAT activity.

Another apoA-I PTM, detected in plasma samples of patients with type-1 diabetes, which reduces LCAT catalytic efficiency and disrupts cholesterol transport, is methionine oxidation (Shao et al., 2006, 2008; Brock et al., 2008). It is interesting to note that oxidized methionine residues alter the conformation of N_t_ and C_t_ when apoA-I is either lipid free or lipid-bound, and limited proteolysis experiments suggest that, following methionine oxidation, the N_t_ becomes highly solvent accessible and the posttranslational modified lipid-bound apoA-I is cleaved at Y_192_ (Ji and Jonas, 1995; Roberts et al., 1997; Calabresi et al., 2001). Another PTM of apoA-I detected in atherosclerotic plaque, that reduces the capacity of lipid bound apoA-I to activate LCAT, is the MPO-mediated nitration of tyrosine at Tyr_166_ (Peng et al., 2005; Zheng et al., 2005; Wu et al., 2007; DiDonato et al., 2014). DiDonato et al. showed that the mutant Tyr${}_{166}^{\text{NO}2}$-apoA-I reduces the capacity of lipid bound apoA-I to activate LCAT *in vitro*, however, the impact on HDL phenotype *in vivo* is not yet understood (DiDonato et al., 2014).

In conclusion, PTMs of apoA-I resulting from HDL interaction with several biological agents (either metabolites or enzymes) diminish apoA-I's ability to exchange between lipid-free/lipid-poor and lipid-bound (HDL) states, a critical feature indispensable for its participation in the RCT cycle (Cavigiolio et al., 2010; DiDonato et al., 2013).

## Restoring HDL functionality

Research carried out over the last decade indicates that the effects of HDL dysfunction can be ameliorated to some extent by therapies with statins and niacin, drugs that can restore the activity of some functional facets of dysfunctional HDL (Lüscher et al., 2014; Rosenson et al., 2016). This section briefly reviews some of the many clinical studies carried out so far to test the effects of several drugs on HDL phenotype and RCT overall.

Khera et al. reported that plasma from patients undergoing statin therapy can recover cholesterol efflux from macrophages (Khera et al., 2011; Miyamoto-Sasaki et al., 2013), for example, plasma from patients with type IIb hyperlipoproteinaemia treated with atorvastatin elevates cholesterol efflux from Fu5AH hepatoma cells (Guerin et al., 2002). Similarly, plasma from dyslipidaemic patients given pitavastatin, increases the efflux of cholesterol from THP-1 macrophages, the PL content of HDL, and the activity of PON1 enzyme (Miyamoto-Sasaki et al., 2013). As a mechanism of action it was suggested that statins might interfere with ABCA1-mediated cholesterol efflux pathways (Niesor et al., 2015).

The effect of niacin on various HDL functions was reported to be uneven, but it was found to elevate HDL plasma levels and reduce TGs. For example, niacin therapy promotes cholesterol efflux from THP-1 macrophages treated with serum HDL (Yvan-Charvet et al., 2010), but has no effect in statin-treated patients (Khera et al., 2013). In another study, niacin therapy in patients with type 2 diabetes restored HDL capacity to increase nitric oxide production, facilitate endothelial healing, and decrease oxidative stress (Sorrentino et al., 2010).

Another category of drugs used for restoring the physiological functions of dysfunctional HDL is that of inhibitors of cholesteryl ester transfer protein (CETP). Clinical studies revealed that CETP inhibitors can be effective in increasing HDL plasma levels, but questions about their toxicity and success in restoring HDL function linger. For example, torcetrapib was found to have high toxicity (Barter et al., 2007), however its use in patients treated with atorvastatin enhances cholesterol efflux along pathways involving the SCARB1 and ABCG1 receptors (Catalano et al., 2009). On the other hand, a trial using dalcetrapib, a drug that modestly elevates HDL plasma levels, was discontinued for lack of progress in patients with acute coronary syndrome (ACS; Ballantyne et al., 2012), so researchers concluded that dalcetrapib has either an insignificant effect on restoring HDL function or more dysfunctional HDL is produced during treatment. Newer CETP inhibitors that increase the levels of plasma HDL and reduce plasma LDL significantly are anacetrapib and evacetrapib (Cannon et al., 2010; Nicholls et al., 2011), but their efficacy in remedying HDL function awaits extensive trials.

## Conclusion

This review on recent progress on lipid free apoA-I and HDL structure and function focuses on theoretical models of lipid free apoA-I, on one hand, and models of HDL derived from SANS with contrast variation, on the other hand. This presentation was motivated by the need to complement recent reviews of the field that overlook the low resolution structures of nHDL and sHDL obtained from SANS with contrast variation. The latter approach has numerous advantageous features: e.g., is non-perturbing due to the use of neutrons with low energy, can visualize the protein and lipid shapes separately by using the contrast variation technique, and captures the average conformation of the particle in solution. While the jury is out there on which models of nHDL and sHDL best describe the structural features of these lipoproteins, the SANS with contrast variation measurements performed on nHDL and sHDL are direct experimental observations and the only direct visualization of these particles at a resolution of 15–20 Å.

In addition to early and more recent structural data on lipid free and lipid bound apoA-I, this review discusses many naturally occurring and bioengineered mutations, and PTMs of apoA-I and their impact on the structure and the function of HDL. Finally, recent progress in the use of drug therapy for restoring the physiological functions of dysfunctional HDL was briefly recounted.

### Conflict of interest statement

The author declares that the research was conducted in the absence of any commercial or financial relationships that could be construed as a potential conflict of interest.
